# Altered acylcarnitine metabolism and inflexible mitochondrial fuel utilization characterize the loss of neonatal myocardial regeneration capacity

**DOI:** 10.1038/s12276-023-00967-5

**Published:** 2023-04-03

**Authors:** E. Kankuri, P. Finckenberg, J. Leinonen, M. Tarkia, S. Björk, J. Purhonen, J. Kallijärvi, M. Kankainen, R. Soliymani, M. Lalowski, E. Mervaala

**Affiliations:** 1 Department of Pharmacology, Faculty of Medicine, University of Helsinki, Helsinki, Finland; 2 Folkhälsan Research Center, Helsinki, Finland; 3 Stem Cells and Metabolism Research Program, Faculty of Medicine, University of Helsinki, Helsinki, Finland; 4 Medical and Clinical Genetics, Faculty of Medicine, University of Helsinki, Helsinki University Hospital, Helsinki, Finland; 5 Translational Immunology Research Program and Department of Clinical Chemistry, Faculty of Medicine, University of Helsinki, Helsinki, Finland; 6 Helsinki Institute of Life Science (HiLIFE), Meilahti Clinical Proteomics Core Facility, Department of Biochemistry and Developmental Biology, Faculty of Medicine, University of Helsinki, Helsinki, Finland; 7 Department of Gene Expression, Institute of Molecular Biology and Biotechnology, Faculty of Biology, Adam Mickiewicz University, Poznań, Poland

**Keywords:** Translational research, Heart failure

## Abstract

Myocardial regeneration capacity declines during the first week after birth, and this decline is linked to adaptation to oxidative metabolism. Utilizing this regenerative window, we characterized the metabolic changes in myocardial injury in 1-day-old regeneration-competent and 7-day-old regeneration-compromised mice. The mice were either sham-operated or received left anterior descending coronary artery ligation to induce myocardial infarction (MI) and acute ischemic heart failure. Myocardial samples were collected 21 days after operations for metabolomic, transcriptomic and proteomic analyses. Phenotypic characterizations were carried out using echocardiography, histology and mitochondrial structural and functional assessments. In both groups, MI induced an early decline in cardiac function that persisted in the regeneration-compromised mice over time. By integrating the findings from metabolomic, transcriptomic and proteomic examinations, we linked regeneration failure to the accumulation of long-chain acylcarnitines and insufficient metabolic capacity for fatty acid beta-oxidation. Decreased expression of the redox-sensitive mitochondrial Slc25a20 carnitine-acylcarnitine translocase together with a decreased reduced:oxidized glutathione ratio in the myocardium in the regeneration-compromised mice pointed to a defect in the redox-sensitive acylcarnitine transport to the mitochondrial matrix. Rather than a forced shift from the preferred adult myocardial oxidative fuel source, our results suggest the facilitation of mitochondrial fatty acid transport and improvement of the beta-oxidation pathway as a means to overcome the metabolic barrier for repair and regeneration in adult mammals after MI and heart failure.

## Heart disease: Lessons from newborns about regeneration

Insights into the ability of heart muscle to only regenerate within the first few days after birth are suggesting options for encouraging regeneration in later life, which might allow heart damage to be reversed. Researchers in Finland led by Eero Mervaala at the University of Helsinki examined the metabolic changes in mice that lead to the loss of the heart tissue’s regenerative capacity soon after birth. Their work implicated a reduced ability to metabolize fatty acids and the accumulation of molecules involved in energy generation called acylcarnitines that stimulate mitochondrial reactive oxygen species production. These results and other molecular details suggest that stimulating mitochondria, the energy-processing centers of cells, to use fatty acids more efficiently as an energy source might help cardiac repair by providing a metabolic state closer to that found in newborns.

## Introduction

During the first week after birth, the potential for myocardial regeneration wanes rapidly in mammals^1^. This period is characterized by a metabolic shift from glycolysis to fatty acid oxidation^2^ and increased formation of mitochondrial reactive oxygen species (ROS), contributing to cardiomyocyte proliferation arrest^3^. The increased understanding of mammalian regeneration capacity at the molecular level^4^ has inspired hope that new therapeutic approaches will be developed to functionally restore damaged and failing human hearts^5^. Although it has been established that major physiological changes accompany the postnatal decline in myocardial regeneration capacity, the associated metabolic changes and shift in metabolic responses toward injury and repair have received less attention.

Major cardiac injuries such as large myocardial infarction (MI) lead to systolic heart failure because of cardiomyocyte loss and fibrotic scar formation. The cardiac-function-restricting fibrotic repair of the adult heart muscle after MI contributes to the development of heart failure (HF) with high five-year mortality. Since research on neonatal hearts’ ability to functionally recover from ischemic damage has thus far mainly focused on cardiomyocyte proliferation^6,7^, we set out to characterize the metabolic changes between the myocardia of regeneration-competent and regeneration-compromised mice and the functional, structural, and metabolic changes induced by myocardial injury in both groups. Metabolic changes in stem and cancer cells suggest tight links among metabolism, cell cycle progression and tissue regeneration^8^, but these links remain poorly understood in relation to myocardial regeneration competence. To provide a therapeutically translatable view, we integrated results from metabolomic, transcriptomic and proteomic analyses to gain comprehensive insight into processes either limiting or driving cardiac regeneration.

For short periods, the adult heart can flexibly shift among carbohydrates, fatty acids, ketone bodies, and amino acids as fuel sources^9^. Ultimately, the metabolism of all these fuel sources converges at the level of mitochondrial oxidative phosphorylation, which accounts for more than 90% of the adult heart’s energy production in the form of ATP^10^. When oxygen is available, fatty acid oxidation (FAO) is the most efficient route for generating ATP^11^, and adult cardiomyocytes rely on this metabolic pathway to generate 70–80% of their energy requirement. Neonatal hearts, on the other hand, have a high rate of glycolysis that is tightly linked to increased cardiomyocyte proliferation^10^. After birth, the change from a low-oxygen to a normoxic environment promotes a rapid increase in mitochondrial biogenesis, which is critical for successful transition to oxidative metabolism to support the increasing energy requirements for efficient blood circulation^12^. This change relies on peroxisome proliferator activated receptor-gamma coactivator-1-alpha (Ppargc1a, Pgc-1α), a transcriptional coactivator that critically regulates several cardiac metabolic and functional cardiac maturation processes^13^.

In patients with HF, cardiac metabolism and mitochondrial function are dysregulated^10,14^. Furthermore, metabolomic analyses have identified increased circulating concentrations of long-chain acylcarnitines that correlate with declining left ventricular ejection fraction (LVEF) and contribute to arrhythmias^15,16^. Acylcarnitines have also been connected to cardiac and mitochondrial damage^17,18^. These changes have been linked upstream to abnormalities at the level of the key cardiac metabolic regulator Pgc-1α^19^.

Our aim was to investigate the cardiac metabolic and transcriptional changes after MI in both 1-day-old and 7-day-old mice, which are in the key time window during which regeneration competence is lost. After induction of MI, the groups were followed up for 3 weeks in a regular normoxic environment, which is known to induce and maintain oxidative metabolism. Moreover, to ensure the success of MI induction, we assessed early transcriptomic changes 3 days after MI and evaluated functional and structural myocardial damage by histology and echocardiography over the follow-up time. Our comparative differential analysis of myocardial tissue identified increased amounts of ketone bodies and accumulation of acylcarnitines at the level of the mitochondrial carnitine shuttle after myocardial ischemic damage in regeneration-compromised mice.

Our results provide metabolomic characterization of myocardial regeneration failure occurring early after birth and tightly link the fixation of mature myocardium on fatty acid metabolism to acylcarnitine accumulation and tissue damage after MI. We also present a conceptual metabolomic- and transcriptomic-based rationale for the targeted design and development of future therapies aimed at modulating mitochondrial bioenergetics for functional myocardial recovery after irreversible damage in patients with ischemic heart disease, MI, and HF.

## Materials and methods

### Animals

One- (P1) and seven-day (P7) old male Hsd:ICR(CD-1) mice were divided into four groups subjected to a follow-up period of 21 days as follows: (1) the 1-day-old sham-operated (1d-SHAM + 21d) group, (2) the MI-induced 1-day-old group (1d-MI + 21d), (3) the 7-day-old sham-operated group (7d-SHAM + 21d), and (4) the MI-induced 7-day-old group (7d-MI + 21d). A complementary study was conducted where the follow-up period was shortened to three days (1d-SHAM + 3, 1d-MI + 3, 7d-SHAM + 3, 7d-MI + 3). All animal experiments were carried out according to the European Community guidelines for the use of experimental animals and approved by the Finnish National Animal Experiment Board (permission numbers ESAVI/8054/04.10.07/2016, ESAVI/31851/2019).

### Operations

The on-ice neonatal mouse MI model has been previously reported in detail by Mahmoud et al.^20^. A detailed description is provided in the Supplementary Materials.

### Echocardiography

Transthoracic echocardiography was performed on postoperative days 1, 3, and 21 by using a Vevo 2100 ultrasound with an MS700 (bandwidth 30–70 MHz) linear array transducer (VisualSonics Inc., Toronto, Canada). The images were stored and analyzed using Vevo LAB 1.7.1 software (VisualSonics Inc., Toronto, Canada). The chamber diameter was measured from the 2D-guided parasternal short axis M-mode images from which the ejection fraction (EF) and fractional shortening (FS) were calculated.

### Euthanasia and sample collection

At the end of the follow-up period (3 days or 21 days), the animals were weighed, anesthetized with isoflurane, and decapitated. The hearts were exposed, collected, and weighed. Whole hearts for histology were fixed in formalin, embedded in paraffin, and processed into hematoxylin and eosin (HE)-stained sections with routine protocols. Samples (~1 mm^3^) for transmission electron microscopy were collected from the noninfarcted area (remote zone) of the left ventricular wall, fixed in 2.5% glutaraldehyde in 100 mM sodium-phosphate buffer and subsequently embedded in resin by using standard protocols. Samples for transcriptomic, proteomic and metabolomic analyses were frozen in liquid nitrogen and stored at −80 °C until analysis. Following sample collection, the sex of the pups was verified by gonadal examination.

### High-resolution respirometry

The rate of oxidative phosphorylation (OXPHOS) was measured using an Oxygraph-2k instrument (OROBOROS Instruments Corp., Innsbruck, Austria), as we have previously described^2^. A detailed description is provided in the Supplementary Materials.

### Whole-mount immunostaining and volumetric microscopy

For whole-mount immunostaining, the animals were perfused with saline to remove excess blood and then perfusion-fixed with 10% paraformaldehyde. The hearts were removed and kept in 10% paraformaldehyde solution for 24 h until stored in methanol. After immunolabeling and tissue clearing, the 3D-reconstructed images were analyzed for volumetric infarction damage. A detailed description is provided in the Supplementary Materials.

### General histology and mitochondrial ultrastructure

The histology of the myocardial samples was evaluated in a blinded fashion with conventional light microscopy. In brief, the histopathology of the arteries and cardiac muscle tissue was examined. A detailed description is provided in the Supplementary Materials.

### Global metabolomic analysis

Metabolomic analysis was carried out as previously described^2^. Hearts from the sham and MI-treated groups collected after a 21-day follow-up period were shipped to Metabolon, Inc. (Durham, NC, USA), where they were stored at −80 °C until analysis. Sample preparation, quality control, UPLC‒MS/MS and data analysis were all carried out by Metabolon, Inc. A detailed description is provided in the Supplementary Materials.

### Transcriptomic analysis

The cardiac samples stored at −80 °C for whole-transcriptome and miRNA analysis were processed with a Precellys 24 homogenizer with ceramic beads (Bertin Technologies, Montigny-le-Bretonneux, France). RNA was isolated with TRIzol reagent and treated with DNase I. An Agilent Bioanalyzer RNA pico chip (Agilent Technologies Inc., Santa Clara, CA, USA) was used to evaluate the integrity of RNA, and a Qubit RNA kit (Thermo Fisher Scientific Inc., Waltham, MA, USA) was used to quantitate RNA.

### mRNA and miRNA analyses

Detailed descriptions of the sample preparation, NGS and data analysis methods are provided in the Supplementary Materials.

### Proteomic analyses

After homogenization and processing of protein lysates, the samples were analyzed by DIA-HDMS^E^ (data independent acquisition high-definition ion-mobility enabled tandem mass spectrometry), as described previously^2^. A detailed description is provided in the Supplementary Materials.

### Ingenuity pathway analysis (IPA)

Pathway analyses were carried out as described previously^21^. A detailed description is provided in the Supplementary Materials.

### Statistical analyses

For data on phenotypic and electron microscopy characterizations, one-way ANOVA and Tukey’s multiple comparisons test were used. For metabolomics, Metabolon’s statistical workflow utilizing two-way ANOVA contrasts followed by contrasts with Welch’s *t* test was used to identify metabolites that differed significantly between the experimental groups. The results were considered significant at *P* < 0.05. For proteomics and transcriptomics, the details of the statistical workflows are provided in the Supplementary Materials.

## Results

### Myocardial infarction and heart failure phenotypes

**Fig. 1:**
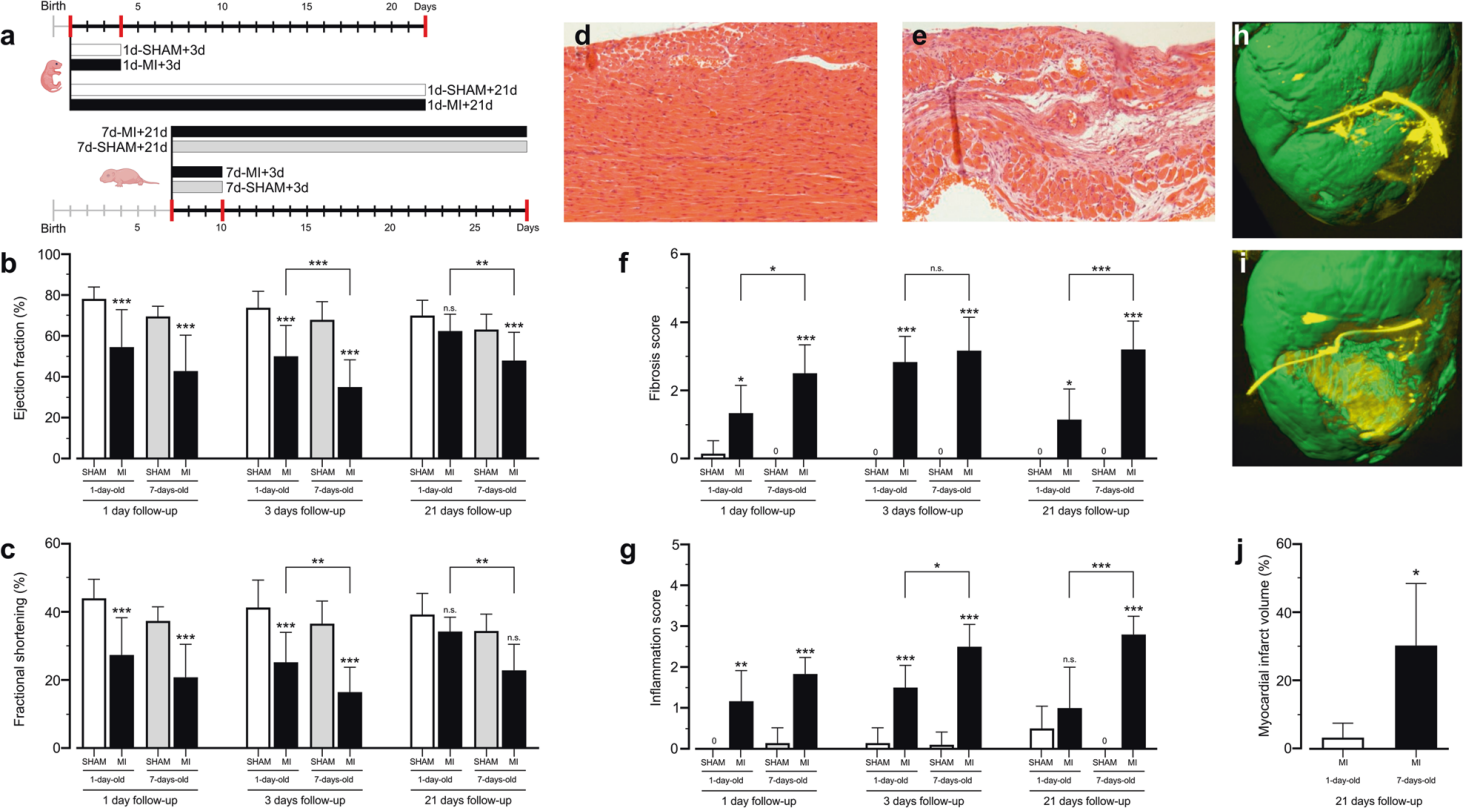
Study outline, cardiac histo-morphological and functional outcomes. **a** Study timeline, interventions and main groups. **b** Left ventricular ejection fraction and **c** fractional shortening in the sham and MI groups at time points 1, 3 and 21 after intervention. **d**, **e** Representative histological images from the (**d**) 1d-MI + 21 and (**e**) 7d-MI + 21 groups. **f**, **g** Histological scoring of (**f**) damage and (**g**) inflammation in the sham and MI groups at time points 1, 3 and 21 after intervention. LAD, left anterior descending coronary artery. **h**, **i** 3D-reconstructed representative iDISCO image of the left ventricle in the (**h**) 1d-MI + 21 and (**i**) 7d-MI + 21 groups. **j** Quantitation of myocardial infarction volume in 1-day-old and 7-day-old animals at the 21-day follow-up. The animals in the 1d-MI + 21 group exhibited small partial fibrotic changes located in the anterior LV wall, whereas large and clear transmural infarct scars were observed in 7d-MI + 21 animals. Histological H&E staining shows the almost unchanged morphology of the 1d-MI + 21 heart compared to the vastly fibrotic 7d-MI + 21 heart. For histopathological analysis, *n* = 6–10, and for echocardiography, *n* = 8–14. For volumetric evaluation, *n* = 4 separate animals. The data are presented as the mean ± SD. *P < 0.05, **P < 0.01, ***P < 0.001 between MI and corresponding sham or as indicated.

Figure 1a shows the study outline. To compare the regenerative functional properties of the myocardium after MI, we assessed cardiac function by echocardiography (Fig. 1b, c). LAD ligation-induced MI caused a similar reduction in ejection fraction and fractional shortening in both regeneration-competent and regeneration-compromised mice during the three days after MI. Three days after MI, the cardiac function of the regeneration-competent mice demonstrated improvement over that of their regeneration-compromised counterparts (Fig. 1b). After 21 days, when the 1-day-old and 7-day-old animals had reached the ages of 22 and 28 days, respectively, the mice in the 1d-MI + 21 group had recovered their cardiac function to the level of control animals. The ejection fraction and fractional shortening in the 7d-MI + 21 group remained lower than those in their respective controls and the 1d-MI + 21 mice.

Although the tissue necrosis and inflammatory response were evident 3 days after the injury, the affected regions of the hearts of the 1d-MI + 21 group demonstrated normal myocardial gross morphology after the 3-week follow-up (Fig. 1d). In contrast, the hearts of the 7d-MI + 21 mice showed major histopathological lesions with vast areas of scar development occupying up to 30% of the left ventricle (7d-MI + 21 30.2 ± 9.1% vs. 1d-MI + 21 3.2 ± 2.1%; *n* = 4; *P* = 0.028; Fig. 1e). Scoring of the histopathological changes (Fig. 1f) demonstrated increases in myocardial necrosis and inflammatory response in both the regeneration-competent and regeneration-compromised groups at 1 d and 3 d time points. On the 21st day of follow-up, the relative level of structural damage observed at 3 d was sustained in the 7d-MI + 21 mice, as the formerly necrotic areas had undergone reparative fibrosis with evident scar tissue. However, the 1d-MI + 21 mice had marked reductions in the visibly affected areas and showed only smaller distinct fibrotic bundles (3.17 ± 0.98 vs. 2.83 ± 0.75, *P* > 0.05 at 3 days and 3.20 ± 0.84 vs. 1.14 ± 0.90, *P* < 0.001 at 21 days). Scoring of the inflammation revealed a persistently increased inflammatory reaction only in the 7d-MI + 21 group. At earlier time points after MI, however, both the 1d-MI + 21 and 7d-MI + 21 groups demonstrated an increased inflammatory response (1.17 ± 0.75 vs. 1.83 ± 0.41, *P* > 0.05). Inflammation remained at a significantly lower level in the 1d-MI + 21 group than in the 7d-MI + 21 group at both 3 days and 21 days of follow-up (1.50 ± 0.55 vs. 2.50 ± 0.55 at 3 days, *P* < 0.05 and 1.00 ± 1.00 vs. 2.80 ± 0.45, *P* < 0.001 at 21 days, Fig. 1g). iDISCO 3D imaging of the myocardium further validated the differences in fibrosis and myocardial damage between the 7d-MI + 21 and 1d-MI + 21 groups (Fig. 1h, i). Supplementary Videos 1 and 2 present the corresponding 3D movies. Volumetric assessment of infarction demonstrated a 9.6-fold larger infarction volume percentage in the 7d-MI + 21 group than in the 1d-MI + 21 group (30.2 ± 18.2% vs. 3.15 ± 4.27%, Fig. 1j).

**Fig. 2:**
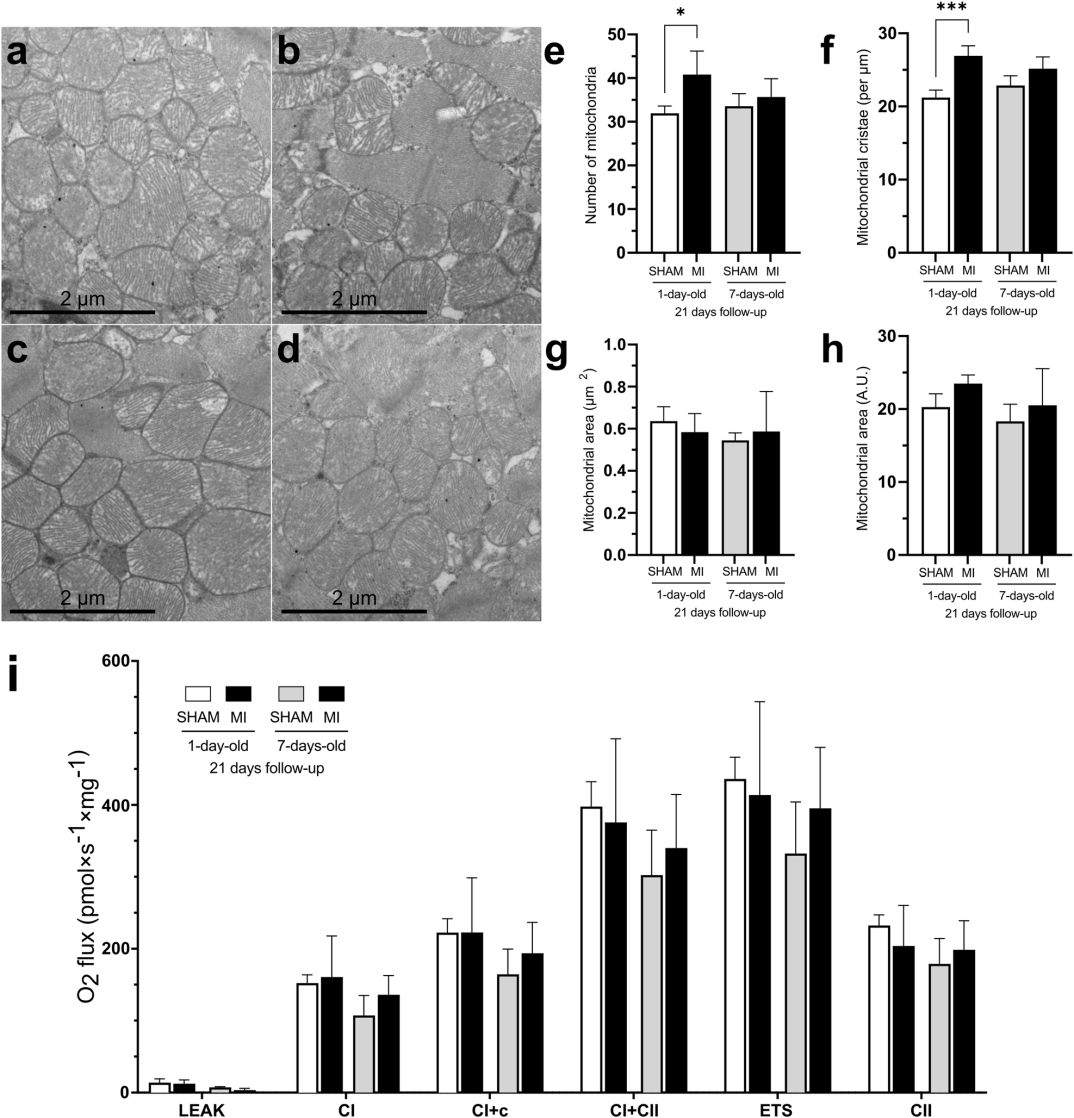
Mitochondrial structure and function. **a**–**d** Transmission electron microscopy images of cardiac mitochondria. **a** 1d-SHAM + 21, **b** 7d-SHAM + 21, **c** 1d-MI + 21, **d** 7d-MI + 21. **e**–**h** Mitochondrial variables: (**e**) number of mitochondria, (**f**) number of cristae, (**g**) volume fraction and (**h**) volume. **i** High-resolution respirometry (HRR) measurement of mitochondrial oxidative phosphorylation component activity in 1d + 21 and 7d + 21 mice. Oxygen consumption rates measured by mitochondrial HRR between groups. For HRR, *n* = 4; for mitochondrial analysis, *n* = 3–4 from a total of 793-1,051 mitochondria/group and from a total of 2023–2722 cristae/group. The data are presented as the mean ± SD. MI myocardial infarction; ETS electron transport system. *P < 0.05, ***P < 0.001 compared between the sham and MI groups.

We next evaluated mitochondrial structure using transmission electron microscopy (TEM) and mitochondrial function using high-resolution respirometry. Because the number of mitochondria and inner membrane cristae is proportionate to the metabolic activity of the cell and because the active cardiomyocytes have large numbers of cristae in their mitochondria, we quantified the size, number, and cristae of the mitochondria in the cardiomyocytes of the study groups (Fig. 2a–d). The number of mitochondria was increased only in the regeneration-competent 1d-MI + 21 group (Fig. 2e). Additionally, the number of mitochondrial cristae was increased in these animals (Fig. 2f). No differences were observed in the sizes of mitochondria between groups (Fig. 2g, h).

We then evaluated myocardial mitochondrial respiration to decipher whether there were changes in the oxidative phosphorylation capacity 21 days after MI. High-resolution respirometry measurements demonstrated no gross differences in OXPHOS or respiratory electron transfer capacities between the age-matched sham and MI groups (Fig. 2i).

### Metabolomics

**Fig. 3:**
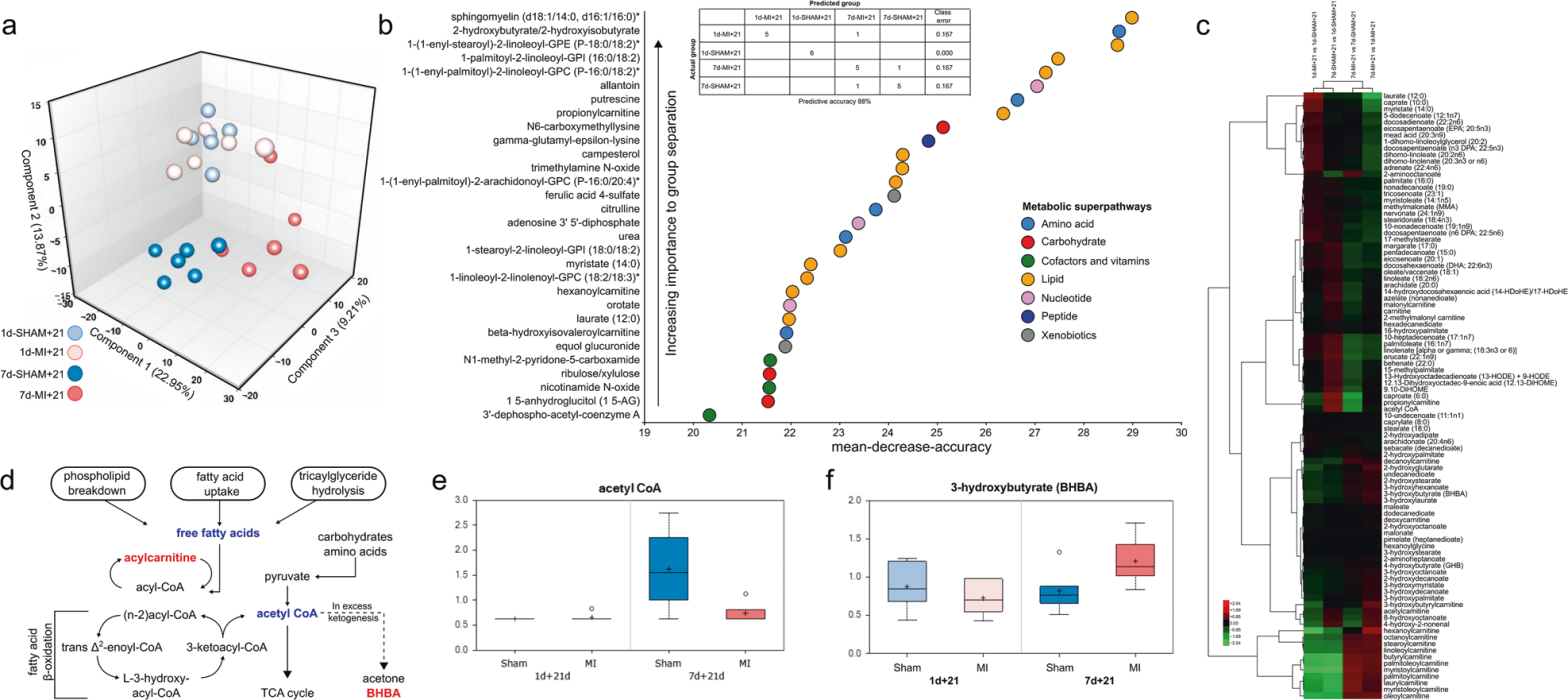
Analysis of the cardiac metabolomes of 1-day-old and 7-day-old mice 21 days after myocardial infarction (MI) or sham operation. **a** Principal component analysis. **b** Random forest prediction of metabolites and their associations with metabolic superpathways and a table of random forest prediction and similarity assessment of groups according to their metabolic profiles. **c** Heatmap of fatty acids and fatty acid metabolites identified in metabolomics analysis. **d** Increased levels of acylcarnitine and defective beta-oxidation resulting in an excess of tissue-level ketogenesis in 7d-MI + 21 mice. Red text indicates an increase, and blue text indicates a decrease, in a comparison between 7d-MI + 21 vs. 1d-MI + 21. Levels of (**e**) acetyl Coenzyme A and (**f**) 3-hydroxybutyrate (BHBA) in different groups. The data are presented as interquartile boxplots showing the median (horizontal line), mean (cross), and minimum–maximum distribution whiskers with outliers (open circles). The metabolite levels in (**e**, **f**) are shown as scaled intensities on the *y* axis. For each group, *n* = 6.

We carried out cardiac tissue metabolomics to identify pathways selectively contributing to the sustained or diminished regenerative ability in 1-day-old and 7-day-old mice after MI. Metabolomic profiling of samples collected at 21 days after sham operation or MI induction (*n* = 6) identified a total of 612 metabolites encompassing eight metabolic superpathways and 86 subpathways. Principal component analysis (PCA) revealed significant time- and treatment-based sample separations of metabolic profiles (Fig. 3a). The samples from 7d-MI + 21 mice clustered separately from their corresponding sham group samples as well as from those of the 1-day-old mice. The 1d-SHAM + 21 and 1d-MI + 21 samples clustered together in the PCA, indicating metabolic restoration at 21 days after MI in the regeneration-competent group. MI in the regeneration-compromised 7-day-old mice, however, significantly altered the myocardial metabolic profile. Overall, the PCA clustering of samples suggested groupwise differences in several unique metabolites.

We then utilized random forest analysis to predict the top metabolic profiles responsible for the groupwise differences. Figure 3b presents the top 30 metabolites based on their importance in separating the metabolic profiles of the groups. Among the top 30 metabolites providing the best separation among groups, 14/30 (43%) were lipid metabolites. The other metabolite classes offered only 3–17% separation ability. The table (Fig. 3b) demonstrates the prediction results.

Supplementary Table 1 lists all identified metabolites and their associated metabolic pathways and superpathways, raw values, group averages and groupwise comparisons. After identification of lipids as the major altered metabolite class, we evaluated the lipid changes in more detail. When comparing the 7d-SHAM + 21 and 7d-MI + 21 samples, we discovered that the 7d-MI + 21d group exhibited significant decreases in most of the long-chain and polyunsaturated fatty acids. However, we found significant increases in most of the long-chain acylcarnitines, which are formed from long-chain fatty acids for their transport into the mitochondria for beta-oxidation (Fig. 3c–d and Supplementary Table 2). This finding underscores the differences in either fatty acid-derived acylcarnitine transport or beta-oxidation rates in regeneration-compromised 7-day-old mice after MI. Contrary to the response observed in 7-day-old mice, most of these alterations were absent or even decreased in the 1d-MI + 21d group compared to its relevant control group, the 1d-SHAM + 21 (Fig. 3c).

At the 21-day follow-up, the mice subjected to surgery one day after birth had lower concentrations of acetyl coenzyme A (acetyl-CoA) than those subjected to surgery on the 7th day after birth (Fig. 3d). MI resulted in a decrease in acetyl-CoA in the 7d-MI + 21 group (Fig. 3e). We also discovered that 3-hydroxybutyrate (BHBA) was elevated 1.48-fold in the 7d-MI + 21d group (Fig. 3f). This water-soluble ketone is typically produced in the liver when glucose levels in the body are low^10,14^. It can be taken up by other tissues, including the heart, and metabolized to acetyl-CoA for fuel. It is thus tempting to speculate that BHBA could be taken up by the hearts of the 7d-MI + 21d mice at increased rates to compensate for impairment in myocardial energy generation.

**Fig. 4:**
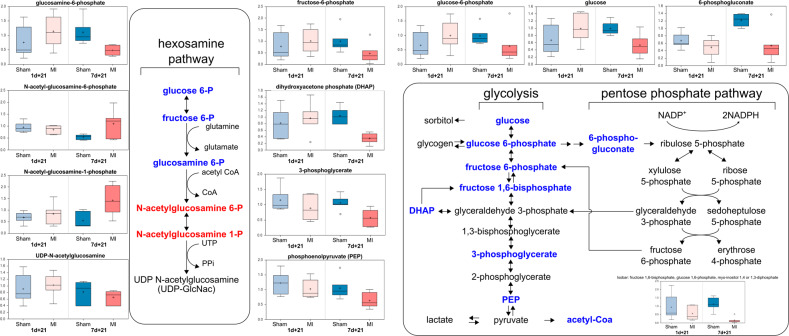
Metabolomic characterization of the hexosamine, glycolysis and pentose phosphate pathways in 1-day-old and 7-day-old mice 21 days after myocardial infarction (MI) or sham operation. The data are presented as interquartile boxplots showing the median (horizontal line), mean (cross), and minimum–maximum distribution whiskers with outliers (open circles). The metabolite levels are displayed as scaled intensities on the *y* axis.

To further elucidate the changes between the regeneration-competent 1-day-old mice and the regeneration-compromised 7-day-old mice after MI, we evaluated the myocardial energy sources and pathways from the metabolomic data. Compared to those of the 7d-SHAM + 21 group, the myocardia of the 7d-MI + 21d mice showed significant decreases in the abundance of glucose and several glucose-derived metabolites (Fig. 4). In essence, all glycolytic intermediates detected were decreased (e.g., glucose 6-phosphate, fructose 6-phosphate, isobaric compound fructose 1,6-diphosphate, dihydroxyacetone phosphate, 3-phosphoglycerate, and phosphoenolpyruvate (PEP)). 6-Phosphogluconate, a metabolite of the pentose phosphate pathway that parallels glycolysis and plays important roles in supporting anabolic growth and antioxidant defenses, was also decreased. Due to the irreversibility of the initial steps in glucose metabolism pathways, the identified metabolite changes strongly suggest altered fuel utilization in the hearts of the regeneration-compromised 7d-MI + 21d mice.

*Tricarboxylic acid (TCA) cycle activity and coenzyme A synthesis*: In addition to the changes noted above, the 7d-MI + 21 hearts also contained lower levels of TCA cycle metabolites than their regeneration-competent counterparts. We discovered decreases in alpha-ketoglutarate, succinylcarnitine, succinate, and fumarate. These changes suggested altered rates of carbon input into the TCA cycle and its upstream regulation due to changes in the glycolytic and beta-oxidation pathways. Decreased amounts of myocardial acetyl-CoA as the end-product of lipid oxidation and pyruvate oxidation in the 7d-MI + 21 group further supported this interpretation (Fig. 3e and Supplementary Fig. 1). Moreover, the regeneration-compromised group also displayed decreases in many of the metabolites, such as pantothenate, phosphopantetheine and 3’-dephosphocoenzyme A, formed in the de novo pathway of coenzyme A synthesis after MI (Supplementary Table 1 and Supplementary Fig. 2).

The 7d-MI + 21 mice showed significant elevations in several plasmalogens, major components of the heart’s phospholipid pool, suggesting deviations in lipid homeostasis, triglyceride and phospholipid synthesis and degradation, which are critical components of myocardial energy production (Supplementary Tables 1 and 3 and Supplementary Fig. 1e). In general, compared to the 7-day-old regeneration-compromised mice after MI, the 1d-MI + 21 mice demonstrated fewer changes in their lipid profiles from those of the time-matched sham control mice. However, we did observe significant changes in this 1d-MI + 21 group in relation to its time-matched sham control group in glycerophospholipid and lysolipid metabolites that were unaltered in the 7d-MI + 21d vs. 7d-SHAM + 21d comparison (Supplementary Table 1).

### Transcriptomics

To obtain insight into acylcarnitine accumulation due to MI in regeneration-compromised mice, we carried out transcriptomic profiling of myocardium samples collected distal to the ischemic insult 21 days after MI or the sham operation (*n* = 3–4).

**Fig. 5:**
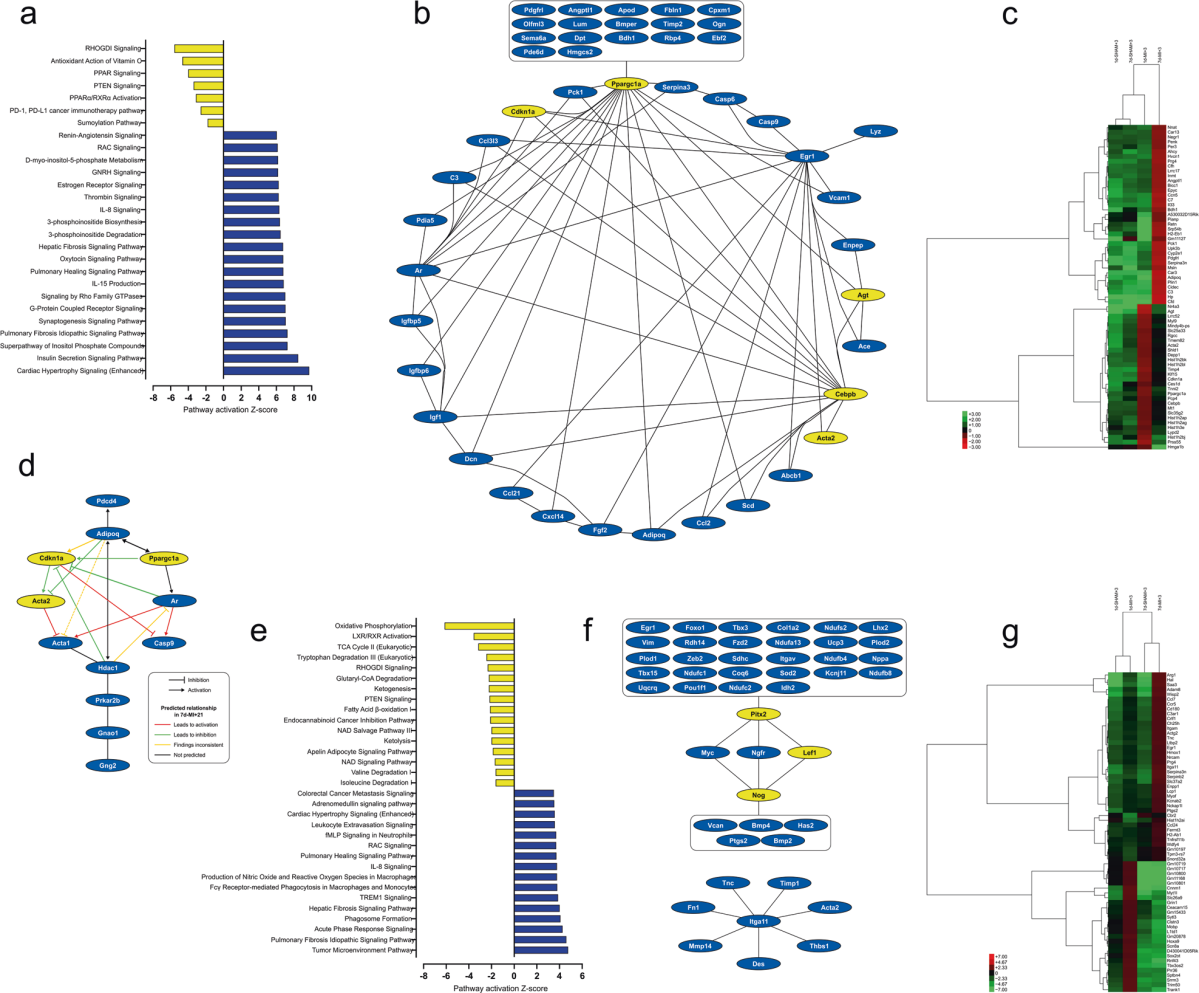
Pathways analysis of transcriptomic data. Pathway analysis, upstream regulatory networks and differentially expressed genes in the 7d-MI and 1d-MI groups at 21 days (**a**–**c**) and 3 days (**e**–**g**). **a**, **e** Canonical pathways that were differentially regulated after myocardial infarction. **b**, **f** Upstream regulatory networks and DEGs. **c**, **g** Heatmap of selected top-scoring DEGs across groups. **d** Network of DEGs in the negative Z value pathways at 21 days. For both the 3-day-old and 7-day-old groups, blue indicates increased values in the 7d-MI groups, and yellow indicates increased values in the 1d-MI groups. The heatmaps show the differentially expressed gene levels in the MI and sham groups of both 1-day-old and 7-day-old mice.

The transcriptome changes predicted increased cardiac hypertrophy and activation of insulin and phosphoinositide signaling pathways in the 7d-MI + 21 group compared to the 1d-MI + 21 group (Fig. 5a). On the other hand, in the 1d-MI + 21 group compared to the 7d-MI + 21 group, the expression of genes in signaling pathways related to RhoGDI, PPAR and PTEN activation was increased (Fig. 5a). Upstream regulator analysis identified a network of differentially expressed genes (DEGs) characterized by decreased expression of *Ppargc1a*, *Cdkn1a*, *Cebpb* and *Agt* in 7d-MI + 21 animals compared to the regeneration-capable 1d-MI + 21 animals (Fig. 5b). The most significantly overexpressed DEGs in the 7d-MI + 21 mice were *H2-Eb1*, *Pianp* and *Cdf* (adipsin), while *Hmga1b*, *Hist1h2bj* and *Agt* were the most overexpressed DEGs in the 1d-MI + 21 group. A heatmap of the top-scoring DEGs at 21 days after MI is shown in Fig. 5c. These results suggest roles for the regulation of inflammation and adipokine induction in the 7d-MI + 21 animals’ myocardial response to MI with decreased expression of *Ppargc1a* and *Cdkn1a*, key regulators of metabolic pathways and cell proliferation, respectively. We then selected those DEGs contributing to pathways with negative z scores (differentially activated in regeneration-competent mice). This analysis (Fig. 5d) further strengthened the association of adipokine (adiponectin, *Adipoq*) regulation by metabolic transcription factors and provided insight into their contribution to the regulation of androgen receptor (*Ar*) expression and apoptosis (*Casp9*, *Pdcd4*).

To provide insight into processes preceding the identified metabolic acylcarnitine accumulation and mitochondrial changes, we analyzed the transcriptome 3 days after induction of MI. Figure 5e shows the pathways differentially activated in the 7d-MI + 3 and 1d-MI + 3 groups. In general, the 7d-MI + 3 mice demonstrated increased gene expression in proinflammatory and acute-phase response signaling, whereas maintenance of metabolic pathways, including oxidative phosphorylation, TCA cycle and fatty acid oxidation, characterized the 1d-MI + 3 group. Upstream regulator analysis identified a network of DEGs and, in regeneration-competent 1d-MI + 3 mice, increased expression of the transcription factors *Pitx2* and *Lef1* as well as increased expression of *Nog*, an inhibitor of transforming growth factor-beta (TGF-beta)-superfamily signaling proteins (Fig. 5f). Figure 5g shows a heatmap of the top DEGs when comparing the 7d-MI + 3 and 1d-MI + 3 groups. In the 7d-MI + 3 mice, the DEGs with the most increased expression included *RP23-100J23.5 (Ofd1*), *Saa3* and *Hal*, suggesting the involvement of primary cilia, inflammation, and histidine degradation in the acute-phase response to MI in regeneration-compromised mice. In contrast, the DEGs with the most increased expression in the regeneration-capable 1d-MI + 3 mice were *Gm10722*, *Gm11168*, and *Gm10801*, all uncharacterized protein-coding transcripts linked in this study with myocardial regeneration after MI.

**Fig. 6:**
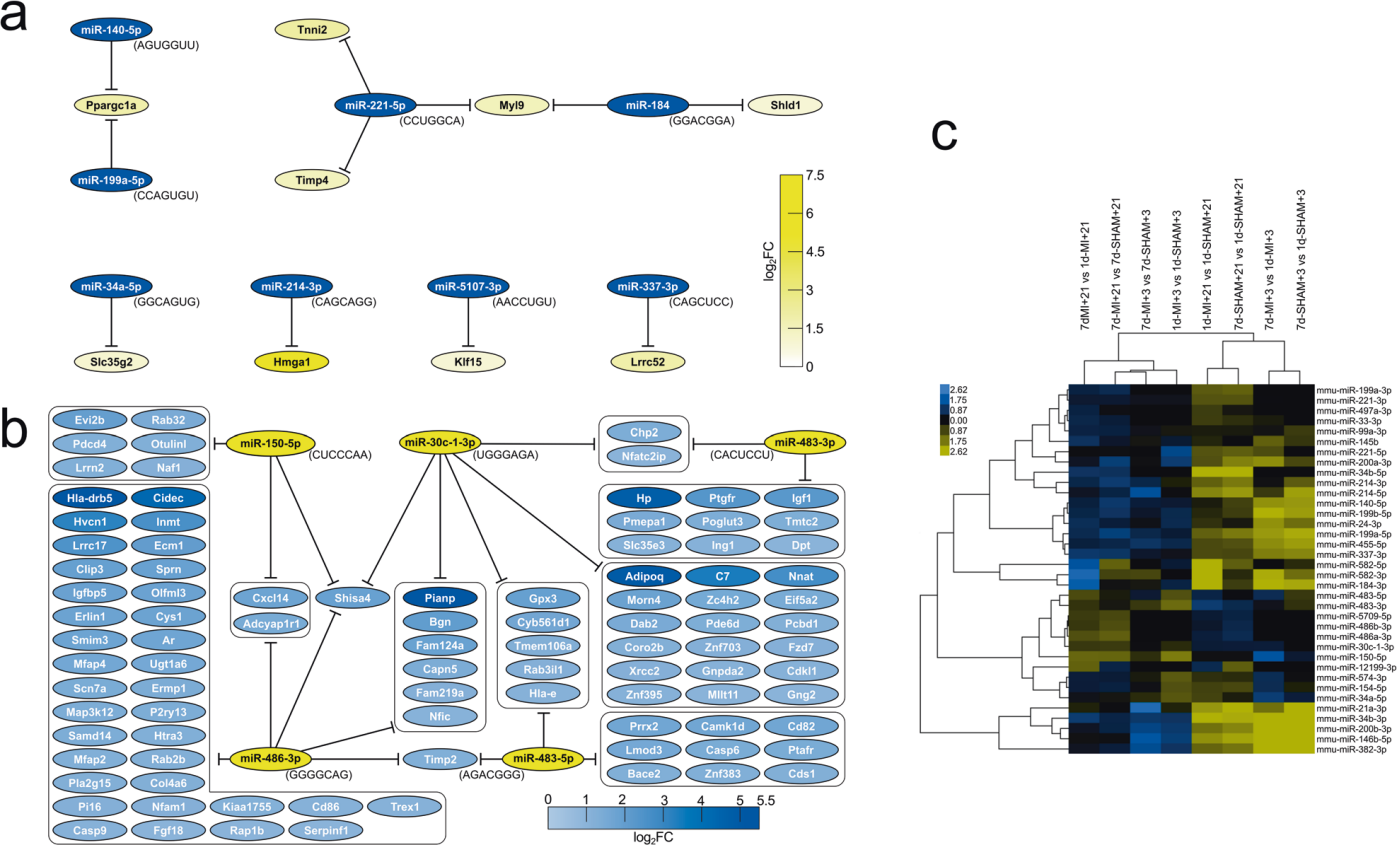
Regulatory miRNAs and their targets differentially expressed in the 7d-MI + 21 vs. 1d-MI + 21 comparison. **a** miRNAs and their DEG targets with high expression in the 7d-MI + 21 group. **b** miRNAs and their DEG targets with high expression in the 1d-MI + 21 group. **c** Heatmap of differentially regulated miRNAs across group comparisons and time points +3 and +21. Blue indicates increased values in the 7d-MI + 21 group. Yellow indicates increased values in the 1d-MI + 21 group.

We then focused on the regulation of DEGs by miRNAs. Utilizing the miRNA target filter in IPA to find oppositely regulated miRNAs and their targets, in 7d-MI + 21 mice, we discovered differentially increased miRNAs, including miR-140-50 and miR-199a-5p targeting *Ppargc1a*, miR-214-3p targeting *Hmga1*, and miR-5107-3p targeting *Klf15* (Fig. 6a). Among the miRNAs with selective increased expression distinctively in 1d-MI + 21 mice, we found a network of miRNAs positively associated with regeneration competence and suppressing an array of DEGs that, in turn, had increased expression in regeneration-compromised mice (Fig. 6b). Of these, miR-150-5p, miR-30c-1-3p and miR-486a/b also had decreased expression when comparing the 7d-MI + 21 and 7d-SHAM + 21 groups (Fig. 6c). Given the high expression of adipokines in regeneration-compromised mice, it is of interest that adiponectin (*Adipoq*) is regulated by miR-30c-1-3p (Fig. 6b). Figure 6c shows the time course of differential miRNA expression at the 21-day follow-up across comparisons at 3 and 21 days.

**Fig. 7:**
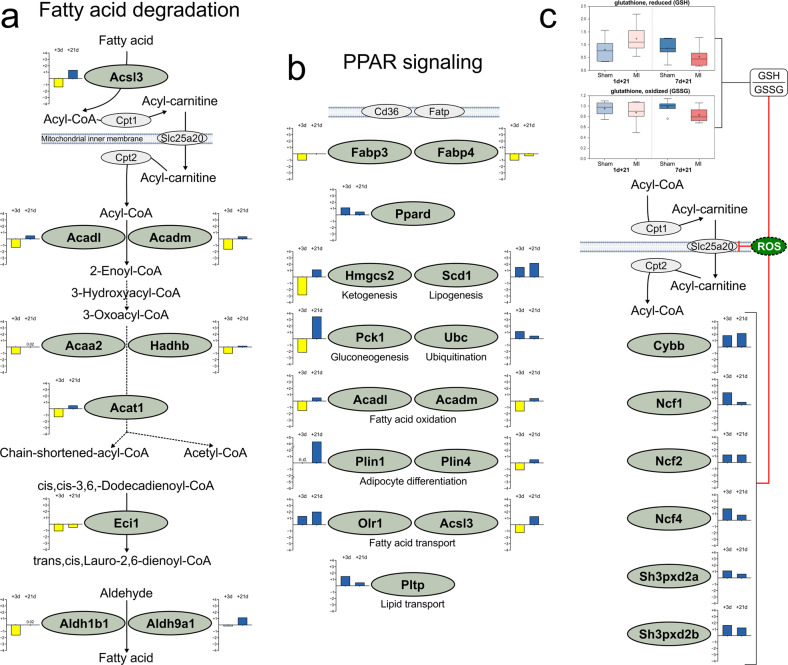
Comparison of gene expression at 3 and 21 days after myocardial infarction according to KEGG pathways. **a** Fatty acid degradation (KEGG pathway mmu00071) and **b** PPAR signaling (KEGG pathway mmu03320). **c** Expression of differentially expressed genes in the superoxide anion-generation Gene Ontology Biological Process (GO:42554) category, metabolomics assessment of the ratio of reduced glutathione to oxidized glutathione, and their suggested contributions to the inhibition of *Slc25a20* (carnitine acylcarnitine transferase, *Cact*) through reactive oxygen species (ROS). Blue indicates increased expression and yellow indicates decreased expression in 7d-MI + 3 and 7d-MI + 21 animals as compared to the 1d-MI + 3 and 1d-MI + 21 animals, respectively. n.d. not detected.

To determine a rationale for the increased acylcarnitine accumulation in 7d-MI + 21 mice, we focused on the KEGG pathways for fatty acid oxidation and PPAR signaling, the regulation of which we previously identified via metabolic and transcriptomic analysis to be key for driving myocardial regeneration. Figure 7a, b compiles these pathways and demonstrate an early critically wide suppression of gene expression in both pathways with only partial recovery at 21 days of follow-up. This result further supports the finding that fatty acid metabolism is profoundly impaired in regeneration-compromised mice compared to regeneration-capable 1-day-old mice. Furthermore, we suggest that impairment at the level of carnitine/acylcarnitine translocase (*Cact*, *Slc25a20*) further contributes to the cellular and tissue accumulation of acylcarnitines in myocardial regeneration failure due to increased production of reactive oxygen species, as evidenced by the persistent increase in superoxide-generation-associated DEGs and reduced antioxidative potential reflected by a decreased reduced:oxidized glutathione ratio (Fig. 7c).

### Label-free proteomic profiling

Proteomic analysis quantified a total of 964 proteins, of which 180 (19%) were significantly differentially expressed (DEPs, FC = > 1.5, *P* < 0.05, *n* = 4) between the 7d-MI + 21 and 1d-MI + 21 groups. We discovered that the relative protein abundance of Slc25a20 carnitine-acylcarnitine translocase was significantly reduced in the 7d-MI + 21 group, potentially contributing to the accumulation of acylcarnitines via decreased shuttling of these fatty acid metabolism intermediates to mitochondria for beta-oxidation after MI in regeneration-compromised mice (Fig. 7a, c and Supplementary Fig. 3). Sixteen DEPs (9%) were also differentially expressed at the transcript level. Among these, 7 DEPs (44%) displayed a positive correlation in their expression directionality with the transcriptomic data. For example, the most upregulated protein in the 7d-MI + 21 regeneration-compromised group was reticulon-2 (Rtn2), which is associated with endoplasmic reticulum stress, poor ventricular function, and heart failure^22^. Aurora kinase B (AurkB) was expressed at a significantly elevated level in these animals. The activity of AurkB has been linked with fibroblast activation and fibrosis^23^. On the other hand, the most significant DEP in the myocardial regeneration-competent 1d-MI + 21 group was keratin 8 (Krt8), an intermediate filament protein shown to convey stress-induced cardioprotection in heart failure^24^. Another highly expressed DEP in regeneration-competent mice was Rnf213, which regulates cell proliferation and angiogenesis^25^. The heatmap of identified DEPs is shown in Supplementary Fig. 3.

The expression patterns of periostin (Postn) and histone H1.5 (Hist1h1b) correlated best across proteomic and transcriptomic data (Supplementary Table 4). Positive correlation of the inhibitor of nuclear factor kappa-B kinase subunit beta (Ikbkb) at the protein and transcript expression levels further validated the presence of a proinflammatory process in 7d-MI + 21 mice. In 1d-MI + 21 animals, Hist1h1b expression was increased and best correlated across platforms. The protein positively regulates cell cycle progression and proliferation as well as regulates Sirt1 expression and its binding to chromatin^26^.

Supplementary Fig. 4 illustrates qPCR validation comparisons of selected transcripts to transcriptomic data. Supplementary Fig. 5 (oxidative phosphorylation; Supplementary Table 5) and Supplementary Fig. 6 (apoptosis; Supplementary Table 6) present KEGG pathways and the corresponding DEGs from transcriptomic analysis of data at both 3 days and 21 days of follow-up.

### Functional probing of acylcarnitine accumulation

To evaluate mitochondrial function under exposure to a physiologically relevant but relatively high concentration of free palmitoyl-l-carnitine, we performed high-resolution respirometry analysis on beta-oxidation and OXPHOS in isolated cardiac mitochondria from regeneration-competent and regeneration-compromised mice collected 3 days after MI or sham operation. In line with the increasing preference for fatty acid oxidation upon cardiomyocyte maturation, mitochondria from 7d-SHAM + 3 mice demonstrated 1.8-fold higher palmitoyl-l-carnitine-induced phosphorylating respiration than mitochondria from 1d-SHAM + 3 mice (Supplementary Fig. 7a). MI decreased the activity of the isolated cardiac mitochondria in the regeneration-competent animals by 30.6% (succinate substrate step: 24.8 pmol/(s×mg) vs. 17.2 pmol/(s×mg)) and in the regeneration-compromised mice by 56.1%. In contrast to the typical respirometry protocol lacking substrates for beta-oxidation (Fig. 2i), the protocol employed here showed that MI also decreased phosphorylating respiration stimulated by the combination of NADH-generating substrates (malate and glutamate) and succinate in the presence of palmitoyl-l-carnitine. This decrease was more notable in the 7d-MI + 3 mice. The identified alterations in energy metabolism pathways may partly explain the observed functional differences, but another explanation is a differential response to the mitochondrial toxicity of long-chain acylcarnitines and acyl-CoAs^27^. To further probe this possibility within the scope of this study, we assessed the modulation of neonatal heart mitochondrial ROS production by palmitoyl-l-carnitine. Palmitoyl oxidation itself did not induce meaningfully quantifiable ROS production over the background (Supplementary Fig. 7b). Instead, upon succinate oxidation, with reverse electron flow blocked by rotenone, 10 µM palmitoyl-l-carnitine roughly doubled the ROS production in state-4 mitochondria (high membrane potential) (Supplementary Fig. 7c).

## Discussion

The postnatal increase in cardiac workload together with the adaptation to respiration and normoxia requires the heart to dramatically increase its oxidative metabolism and convert from using glycolysis to using more ATP-efficient energy pathways. Oxidative metabolism of fatty acids yields higher amounts of ATP per carbon due to the production of more acetyl-CoA, a metabolite critical to several cellular reactions. Although the adult myocardium has an agile ability to utilize glucose, fatty acids, and ketone bodies (e.g., beta-hydroxybutyrate) for fuel, it readily relies on fatty acid oxidation even under prolonged periods of ischemia^28^. The mitochondrial TCA cycle requires acetyl-CoA as well as anaplerotic resources to function^29^. One key resource in replenishing the TCA cycle intermediates is glucose. In the heart, metabolism of glucose to phosphoenolpyruvate (PEP) followed by conversion of PEP to oxaloacetate through a reversible reaction catalyzed by phosphoenolpyruvate carboxykinase (Pck1) can provide such replenishment. According to our transcriptomic analysis, the expression of *Pck1* was differentially increased by 11-fold in 7-day-old mice, indicating a futile effort to revitalize the TCA cycle intermediates by anaplerosis from PEP.

Due to MI in regeneration-compromised mice, the concentrations of glycolysis, TCA cycle and pentose phosphate pathway metabolites were decreased, indicating widespread metabolic pathway depression^30^. In contrast, ketogenesis and hexosamine biosynthetic pathway metabolites were increased in 7-day-old mice compared with 1-day-old mice after MI. The tissue concentrations of glucose, PEP and acetyl-CoA were also decreased, while those of 3-hydroxybutyrate (BHBA), acylcarnitines, and N-acetylglucosamine-6/1 phosphates were increased, compared to those in regeneration-competent 1-day-old mice. The results suggest that the regeneration-compromised heart fuels itself, at least partly, by the uptake of ketone bodies.

Mitochondrial beta-oxidation requires fatty acid transport to the mitochondrial matrix via the carnitine shuttle. Acyl-CoA is converted into acylcarnitine by carnitine palmitoyltransferase I (Cpt1) and then transported through the mitochondrial inner membrane by carnitine-acylcarnitine translocase (Slc25a20). The mitochondrial enzyme Cpt2 then converts acylcarnitine back to acyl-CoA for subsequent beta-oxidation. Increased concentrations of cellular acylcarnitine are known to contribute to inflammation and cellular stress as well as to reduce glucose uptake^31^. When the key metabolic pathways of uninjured, growing neonatal mouse hearts were characterized using metabolomic and proteomic profiling^2^, metabolomic reprogramming from glycolysis (1-day-old mice) to oxidative phosphorylation (7-day-old mice) was evident in these animals, and we also uncovered significant increases in most medium-chain fatty acids and acylcarnitine occurring over normal postnatal development during the first week after birth. These increases were accompanied by upregulation of multiple enzymes involved in fatty acid beta-oxidation. An increase in the formation of reactive oxygen species and an increase in oxidative stress markers were also evident in 7-day-old mice. It is therefore of utmost importance to use time-matched controls and follow-ups when exploring regenerative ability of the first postnatal week in mice as an experimental model.

Here, we found early transcriptional repression of the enzymes involved in fatty acid beta-oxidation after MI in regeneration-compromised 7-day-old mice, suggesting suppression of this pathway in these animals after ischemic damage. Moreover, dominant increases in several acylcarnitines in these mice further suggest the contribution of altered fatty acid metabolism to regeneration incompetence. One contributing factor to this was likely the decreased myocardial abundance of the Slc25a20 translocase found by quantitative proteomics. Moreover, the carnitine shuttle responsible for transporting fatty acids into mitochondria is sensitive to reactive oxygen species^32^. We found the reduced:oxidized glutathione ratio to be decreased in the regeneration-compromised mice after MI, thus potentially impairing the major cellular antioxidant pool and exerting inhibitory effects of reactive oxygen species on the redox-sensitive Slc25a20 translocase. Interestingly, the genetic deficiency of *SLC25A20* is characterized by cardiomyopathy, decreased cardiac function and arrhythmias in addition to liver dysfunction, skeletal muscle damage and neurologic abnormalities^33^. As fatty acid entry into peroxisomes is independent of such a translocase, peroxisomal beta-oxidation likely occurs and contributes to the various species of acylcarnitine identified in our study.

The number of myocardial mitochondria increases rapidly after birth to meet the high energy demands for maintaining increased cardiac output^13^. Although control over mitochondrial quality and number is dynamic and although quality control is regulated through mitophagy, fusion and fission^34^, upon major injury such as abrupt MI, these processes can be overwhelmed and rendered ineffective. In the present study, in which the samples were collected from the left ventricular “remote zone”, mitochondrial numbers and cristae were increased 21 days after MI in the regeneration-competent (3d) group, associating improved mitochondrial metabolic capacity with regeneration ability in these animals. The results also conform with earlier observations linking the myocardial response to infarction with mitochondrial fission^35^.

However, no significant changes in OXPHOS capacities between MI and sham-operated time-matched groups were observed, suggesting dominance and persistence of a metabolic defect related to fatty acid utilization and accumulation of acylcarnitine upon failure to repair the myocardial damage resulting from MI in 7-day-old mice. The cardiac mitochondria of the regeneration-compromised 7-day-old mice better utilized palmitoylcarnitine as an energy source, as indicated by greater mitochondrial oxygen consumption, than the mitochondria of the regeneration-competent 1-day-old mice, thereby confirming their dedication and preference for fatty acid consumption for energy. This effect was further substantiated by a dramatic drop in palmitoylcarnitine oxidation after MI in the 7d-MI + 3 mice. Cardiac mitochondrial respiration stimulated by palmitoylcarnitine in the 1d-MI + 3 mice was also reduced but to a lesser degree than in 7d-MI + 3 mice, illustrating the differences in mitochondrial fuel preferences already at this early stage of follow-up. Furthermore, our results confirmed the previous observation of long-chain acyl carnitines as inducers of mitochondrial ROS^17^. Taken together with the roles of ROS in the promotion of postnatal cardiomyocyte cell cycle arrest and ROS scavenging to reduce myocardial fibrotic scarring and improve systolic function after ischemic insult 3 weeks after birth^3^, our results support the link between acylcarnitine and increased mitochondrial ROS generation. It is tempting to speculate that the accumulation of long-chain acylcarnitines promotes mitochondrial failure through the production of ROS, which in turn blocks mitochondrial beta-oxidation by inhibiting redox-sensitive Slc25a20, leading to further exacerbation of long-chain acylcarnitine accumulation. The accumulation of long-chain acylcarnitine and acyl-CoA also has other toxic effects on mitochondria, the mechanisms of which remain poorly understood^36^. Furthermore, our results further strengthen the evidence of association(s) between increased expression of genes contributing to myocardial ROS generation after MI in regeneration-compromised mice compared to cardiac regeneration-competent mice. Accumulation of fatty acids can activate several intracellular pathways and has been shown to contribute to fibrosis and drive further metabolic failure^37^. The accumulation of fatty acids as acylcarnitine, as demonstrated in this study after MI and compromised myocardial regeneration, suggests that a committed step toward fatty acid metabolism during the 1-week period after birth is critical to regenerative ability.

In general, mutations compromising the function of enzymes in the fatty acid beta-oxidation pathway cause inherited metabolic diseases^38^. Defective carnitine-acylcarnitine shuttling caused by inherited deficiency of carnitine-acylcarnitine translocase can lead to the development of cardiomyopathy^38,39^. Moreover, accumulation of acylcarnitines is arrhythmogenic and can cause deadly ventricular fibrillations and sudden deaths^38,40^. Importantly, failure in beta-oxidation of long-chain, rather than short- or medium-chain, fatty acids has been reported to more likely lead to the development of cardiomyopathy^38^. We also consulted the FinnGen database and its current 7th data freeze, which consists of 429,200 combined genotype and health registry data from more than 309,000 individuals and a total of 3095 disease endpoints^41^. According to the search (https://r7.finngen.fi/gene/SLC25A20), genetic variations in *SLC25A20* are significantly linked with hypertrophic obstructive cardiomyopathy (odds ratio, OR = 3.4; *P* = 0.0022), heart failure and hypertrophic cardiomyopathy (odds ratio, OR = 6.1, *P* = 0.0029), complications following myocardial infarction (OR = 41, *P* = 0.0049), and nonischemic cardiomyopathy (OR = 49; *P* = 0.0052). These associations clearly link defects in *SLC25A20* to human cardiac diseases, especially cardiomyopathies, as well as complications after myocardial infarction, as now observed in our current multiomic study comparing regeneration-competent and regeneration-compromised newborn mouse hearts after myocardial infarction.

MI increased the expression of adipokines such as *adiponectin* and *adipsin (Cfd)* in 7-day-old mice. In heart failure, increased circulating concentrations of adiponectin have been linked with increased mortality and disease severity^42^, although low concentrations of adiponectin have been associated with several beneficial actions^43^. As cardiac dysfunction is worsened in pressure-overloaded hearts of adiponectin knockout mice^44^, it could be hypothesized that increased expression of *adiponectin*, as found in our study, could be a protective response to metabolic changes—especially defects in fatty acid oxidation and mitochondrial function—leading to cardiac fibrosis instead of repair. High blood concentrations of adipsin correlate with all-cause death and rehospitalization in patients with coronary artery disease^45^. Adipsin is a component of the complement alternative pathway and is expressed in inflammation and heart failure^46^. Our results now link high myocardial expression of *adipsin* with MI, fibrosis, and regeneration failure.

Many of the molecular postnatal changes are dependent on reduced Hif signaling^47^; however, the regulation of Ppargc1α, a key regulator of mitochondrial metabolism and biogenesis, has been shown to be oxygen-dependent but not Hif-dependent^47^. The number of mitochondria can be regulated dynamically through mitochondrial fusion, fission and mitophagy^34^. We found Ppargc1α to be in the center of a transcriptional regulatory network involving CCAAT enhancer binding protein beta (*Cebpb*) and *Klf15*, both of which are known dominant controllers of metabolism^48,49^. All had dominantly increased expression in regeneration-competent mice 21 days after MI, and their decreased expression was associated with metabolic control over fatty acid oxidation and decreased accumulation of acylcarnitines.

MicroRNAs are noncoding RNAs, each regulating the expression of a large target set of mRNAs. They are closely linked to orchestrating cardiovascular (patho-)physiological responses^50^. We used a differential comparison analysis between 7d-MI + 21 and 1d-MI + 21 mice to decipher upstream regulatory networks and miRNAs. Namely, miRNAs mmu-miR-140-5p and mmu-miR-199a-5p showed increased expression in regeneration-compromised 7-day-old mice after MI. MiR-140 also regulates other targets related to the cell cycle, apoptosis, and inflammation^51^, and its inhibition has been shown to reduce damage and apoptosis in the myocardium after ischemia‒reperfusion injury^52^. Knockdown of endogenous miR-199a in miR-199-sponge-transgenic mice has been shown to induce physiological hypertrophy targeting Ppargc1α^53^, while exogenous delivery of miR-199 has been demonstrated to induce uncontrolled repair and cardiomyocyte proliferation^54^. We identified mmu-miR-199a-5p to be differentially expressed between regeneration-competent and regeneration-compromised mice 21 days after induction of MI. As suggested by our data, it is likely that regeneration competence is governed by the combined action of several miRNAs, resulting in further specification of the dominant targets for metabolic control. In fact, there was little overlap between the miRNA profiles at 3 days and 21 days of follow-up. Although networks of miRNAs had similar targets, only one miRNA, mmu-miR-21a-3p, demonstrated a regeneration competence-associated increase at both time points. Mmu-miR-21a-3p has been associated with a cardioprotective role in both ischemic disease and cardiomyopathies^55^.

In summary, our data demonstrate that metabolic defects in fatty acid oxidation leading to myocardial accumulation of acylcarnitine underlie the myocardial inability to regenerate after MI. Our results suggest that acylcarnitine transport to mitochondria is defective at the level of the Slc25a20 translocase, a key component of the carnitine shuttle. Furthermore, we conclude that regeneration failure is a mitochondrial metabolic defect that manifests as impaired fatty acid utilization.

## Supplementary information

Supplementary Material

Supplementary Table 3

Supplementary Table 4

Supplementary Table 5

Supplementary Table 1

Supplementary Table 2

Supplementary Video 1

Supplementary Video 2

Supplementary Table 6
