# Supplementary material for: Altered acylcarnitine metabolism and inflexible mitochondrial fuel utilization characterize the loss of neonatal myocardial regeneration capacity
Source: Exp Mol Med. 2023 Apr 3;55(4):806–17. doi: 10.1038/s12276-023-00967-5 (PMC10167339; doi:10.1038/s12276-023-00967-5)
Supplement: Supplementary file 1 — Supplementary Material [file 12276_2023_967_MOESM1_ESM.pdf]

Altered acylcarnitine metabolism and inflexible mitochondrial fuel utilization characterize the loss of neonatal myocardial regeneration capacity

Kankuri E, Finckenberg P, Leinonen J, Tarkia M, Björk S,

Purhonen J, Kallijärvi J, Kankainen M, Soliymani R, Lalowski M, Mervaala E

Supplementary Material

Supplementary Video 1. iDISCO 3D imaging of the heart 21 days after myocardial infarction in a one-day-old mouse (1d-MI+21 heart_iDISCO.mp4).

Supplementary Video 2. iDISCO 3D imaging of the heart 21 days after myocardial infarction in a seven-day-old mouse (7d-MI+21 heart_iDISCO.mp4).

Supplementary Table 1. Metabolomics results. Please see the separate Excel file: Supplementary Table 1.xlsx

Supplementary Table 2. Selected acylcarnitines and carnitine pathway metabolites from the metabolomics analysis. Please see the separate Excel file: Supplementary Table 2.xlsx

Supplementary Table 3. Differential gene expression ($p<0.05$ at either time point) between 7d-MI and 1d-MI in coenzyme A production and TCA cycle pathways. Please see the separate Excel file: Supplementary Table 3.xlsx

Supplementary Table 4. List of the best correlated differentially expressed proteins and transcripts across 7d-MI+21 and 1d-MI+21 groups. DEP, differentially expressed protein; DEG, differentially expressed gene; FC, fold change. Accession- UniProt accession. Please see the separate Excel file: Supplementary Table 4.xlsx

Supplementary Table 5. Differential comparisons of gene expression at 3 and 21 days after myocardial infarction on the mitochondrial electron transport chain according to the KEGG pathway Oxidative Phosphorylation (mmu00190). Please see the separate Excel file: Supplementary Table 5.xlsx

Supplementary Table 6. Differential comparisons of gene expression at 3 and 21 days after myocardial infarction on apoptosis according to the KEGG pathway Apoptosis (mmu04210). Please see the separate Excel file: Supplementary Table 6.xlsx

The proteomics dataset MSV000088783 is openly accessible online through the Mass Spectrometry Interactive Virtual Environment (MassIVE, https://massive.ucsd.edu).

The transcriptomics dataset GSE198302 can be openly accessed online through the Gene Expression Omnibus (GEO, https://www.ncbi.nlm.nih.gov/geo/).

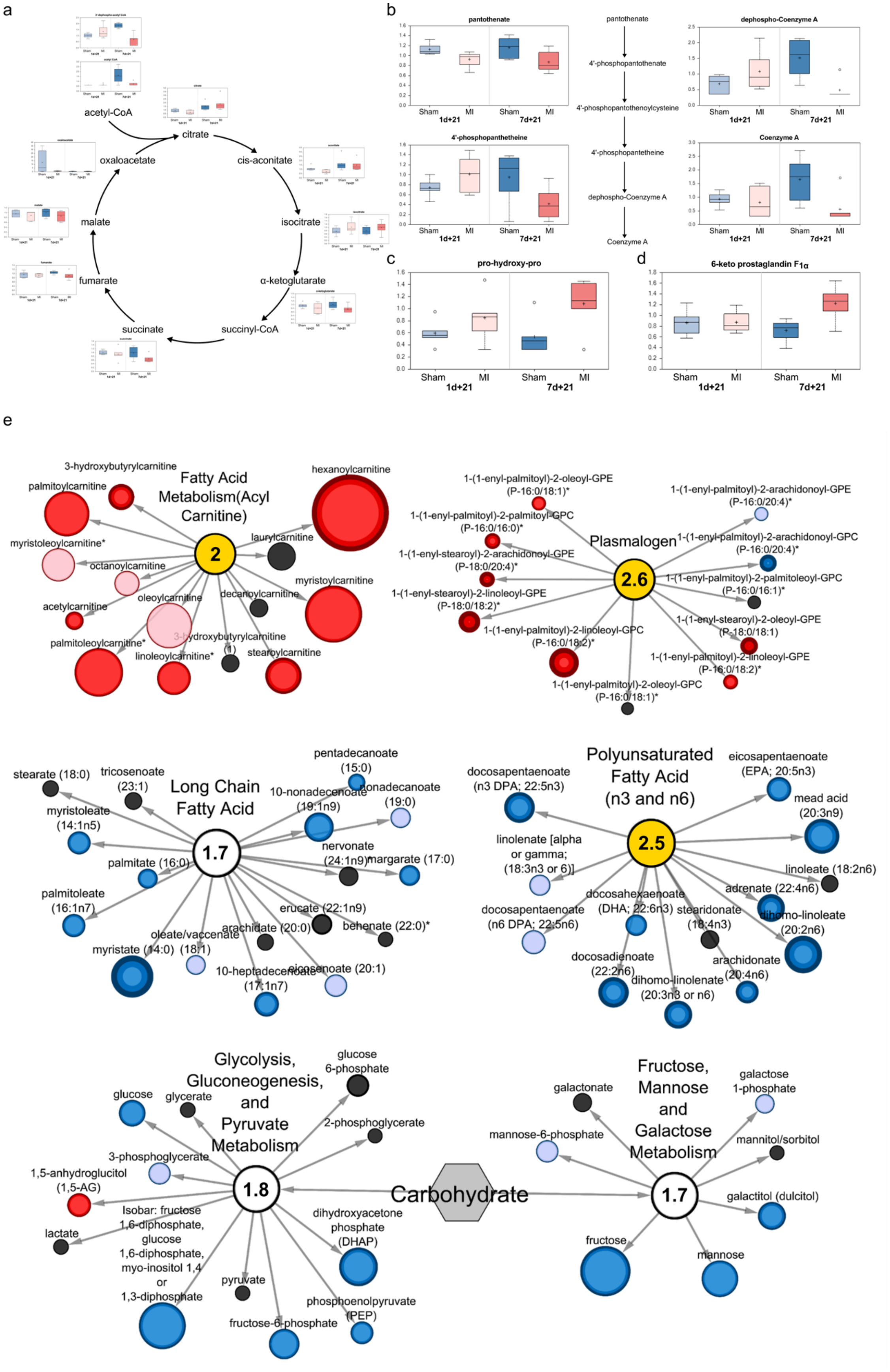

a
acetyl-CoA
citrate
cis-aconitate
isocitrate
α-ketoglutarate
succinyl-CoA
succinate
fumarate
malate
oxaloacetate
b
pantothenate
4'-phosphopantothenate
4'-phosphopantothenoylcysteine
4'-phosphopantetheine
dephospho-Coenzyme A
Coenzyme A
Sham MI Sham MI
1d+21 7d+21
c
pro-hydroxy-pro
d
6-keto prostaglandin F1α
e
Fatty Acid Metabolism(Acyl Carnitine)
2
3-hydroxybutyrylcarnitine
palmitoylcarnitine
hexanoylcarnitine
myristoleoylcarnitine*
octanoylcarnitine
laurylcarnitine
oleoylcarnitine
decanoylcarnitine
myristoylcarnitine
acetylcarnitine
palmitoleoylcarnitine*
3-hydroxybutyrylcarnitine (1)
linoleoylcarnitine*
stearoylcarnitine
Plasmalogen
2.6
1-(1-enyl-palmitoyl)-2-oleoyl-GPE (P-16:0/18:1)*
1-(1-enyl-palmitoyl)-2-arachidonoyl-GPE (P-16:0/20:4)*
1-(1-enyl-palmitoyl)-2-palmitoyl-GPC (P-16:0/16:0)*
1-(1-enyl-palmitoyl)-2-arachidonoyl-GPC (P-16:0/20:4)*
1-(1-enyl-stearoyl)-2-arachidonoyl-GPE (P-18:0/20:4)*
1-(1-enyl-palmitoyl)-2-palmitoleoyl-GPC (P-16:0/16:1)*
1-(1-enyl-stearoyl)-2-linoleoyl-GPE (P-18:0/18:2)*
1-(1-enyl-stearoyl)-2-oleoyl-GPE (P-18:0/18:1)
1-(1-enyl-palmitoyl)-2-linoleoyl-GPC (P-16:0/18:2)*
1-(1-enyl-palmitoyl)-2-linoleoyl-GPE (P-16:0/18:2)*
1-(1-enyl-palmitoyl)-2-oleoyl-GPC (P-16:0/18:1)*
Long Chain Fatty Acid
1.7
stearate (18:0)
tricosenoate (23:1)
pentadecanoate (15:0)
10-nonadecenoate (19:1n9)
nonadecanoate (19:0)
myristoleate (14:1n5)
palmitate (16:0)
nervonate (24:1n9)
margarate (17:0)
palmitoleate (16:1n7)
erucate (22:1n9)
arachidate (20:0)
behenate (22:0)*
oleate/vaccenate (18:1)
myristate (14:0)
10-heptadecenoate (17:1n7)
eicosenoate (20:1)
Polyunsaturated Fatty Acid (n3 and n6)
2.5
docosapentaenoate (n3 DPA; 22:5n3)
eicosapentaenoate (EPA; 20:5n3)
mead acid (20:3n9)
linolenate [alpha or gamma; (18:3n3 or 6)]
linoleate (18:2n6)
docosahexaenoate (DHA; 22:6n3)
adrenate (22:4n6)
docosapentaenoate (n6 DPA; 22:5n6)
stearidonate (18:4n3)
dihomo-linoleate (20:2n6)
docosadienoate (22:2n6)
arachidonate (20:4n6)
dihomo-linolenate (20:3n3 or n6)
Glycolysis, Gluconeogenesis, and Pyruvate Metabolism
1.8
glucose
glycerate
glucose 6-phosphate
2-phosphoglycerate
3-phosphoglycerate
1,5-anhydroglucitol (1,5-AG)
Isobar: fructose 1,6-diphosphate, glucose 1,6-diphosphate, myo-inositol 1,4 or 1,3-diphosphate
lactate
dihydroxyacetone phosphate (DHAP)
pyruvate
phosphoenolpyruvate (PEP)
fructose-6-phosphate
Carbohydrate
Fructose, Mannose and Galactose Metabolism
1.7
galactonate
galactose 1-phosphate
mannose-6-phosphate
mannitol/sorbitol
galactitol (dulcitol)
fructose
mannose

Supplementary Fig. 1. a. Levels of acetyl CoA and tricarboxylic acid (TCA) cycle intermediates 21 days (+21) after myocardial infarction (MI) or sham (SHAM) operation in 1-day-old (1d) and 7-day-old (7d) mice. **b.** Levels of coenzyme A production pathway intermediates at 21 days. **c.** Levels of pro-hydroxy-pro as a marker of fibrotic processes at 21 days. **d.** Level of 6-keto prostaglandin F1alpha as a marker of inflammation at 21 days. Metabolite levels in A-D are visualized as scaled intensities on y-axis. Data are presented as interquartile boxplots showing median (horizontal line), mean (cross), and minimum–maximum distribution whiskers with outliers (open circles). **e.** Selected most significantly altered metabolites in the lipid and carbohydrate metabolic superpathways identified in the comparison of the regeneration-compromised 7d-MI+21 vs regeneration-competent 1d-MI+21 mice. Values in circles represent respective pathway activation z-scores, in yellow pathway enrichment significance with $z \geq 2$; FC, fold change. Gene ID, NCBI Gene name. Please also see the Supplementary Table 3.

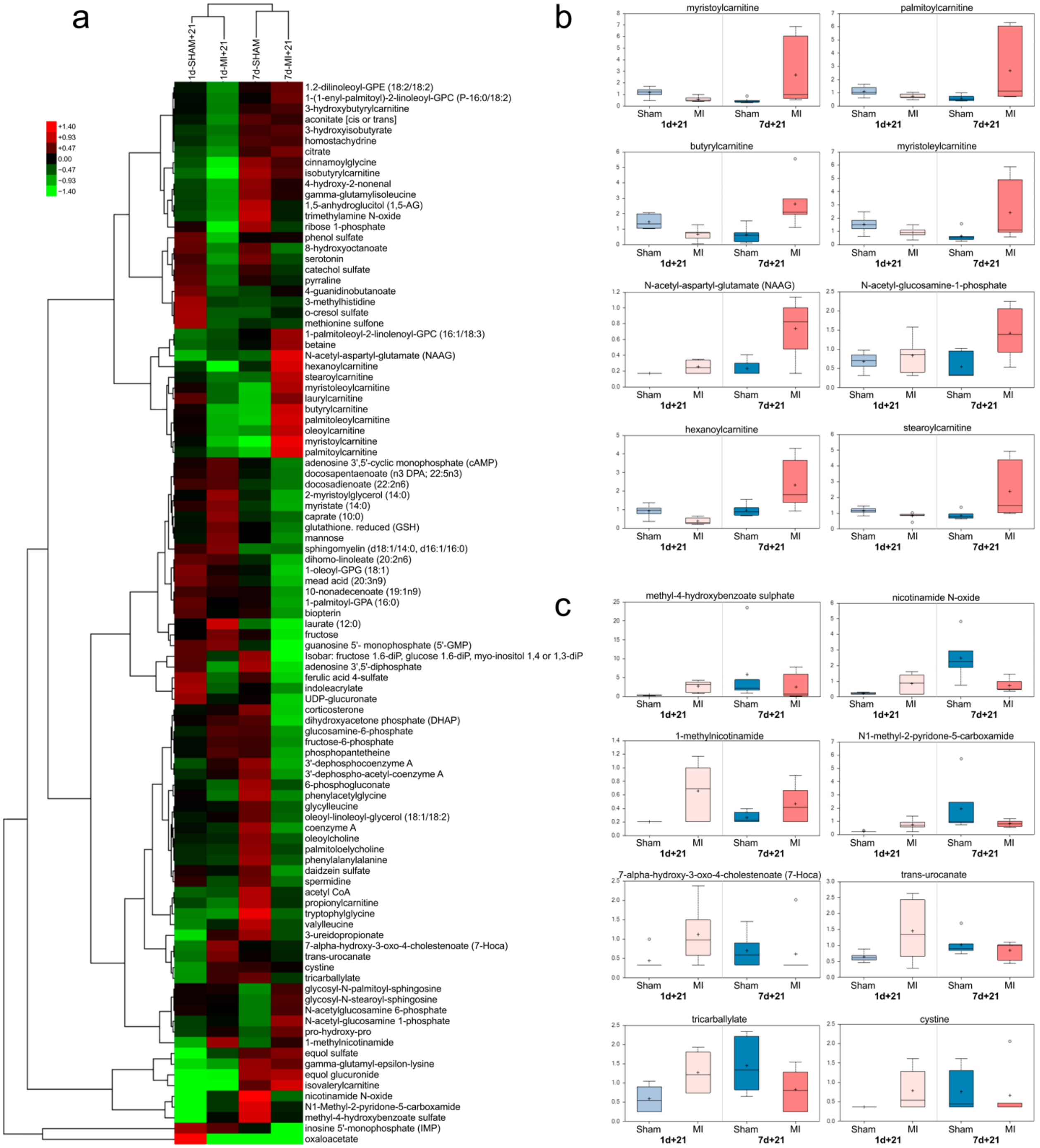

Supplementary Fig. 2. Differentially abundant metabolites (DAMs) between groups. a. Heatmap of DAMs across all groups. **b.** Top 8 DAMs with increased abundance in 7d-MI+21 vs 1d-MI+21 comparison. **c.** Top 8 DAMs with increased abundance in 1d-MI+21 vs 1d-SHAM comparison. Data in **b.–c.** are presented as interquartile boxplots showing median (horizontal line), mean (cross), and minimum–maximum distribution whiskers with outliers (open circles). Metabolite levels in A-D are shown as scaled intensities on y-axis.

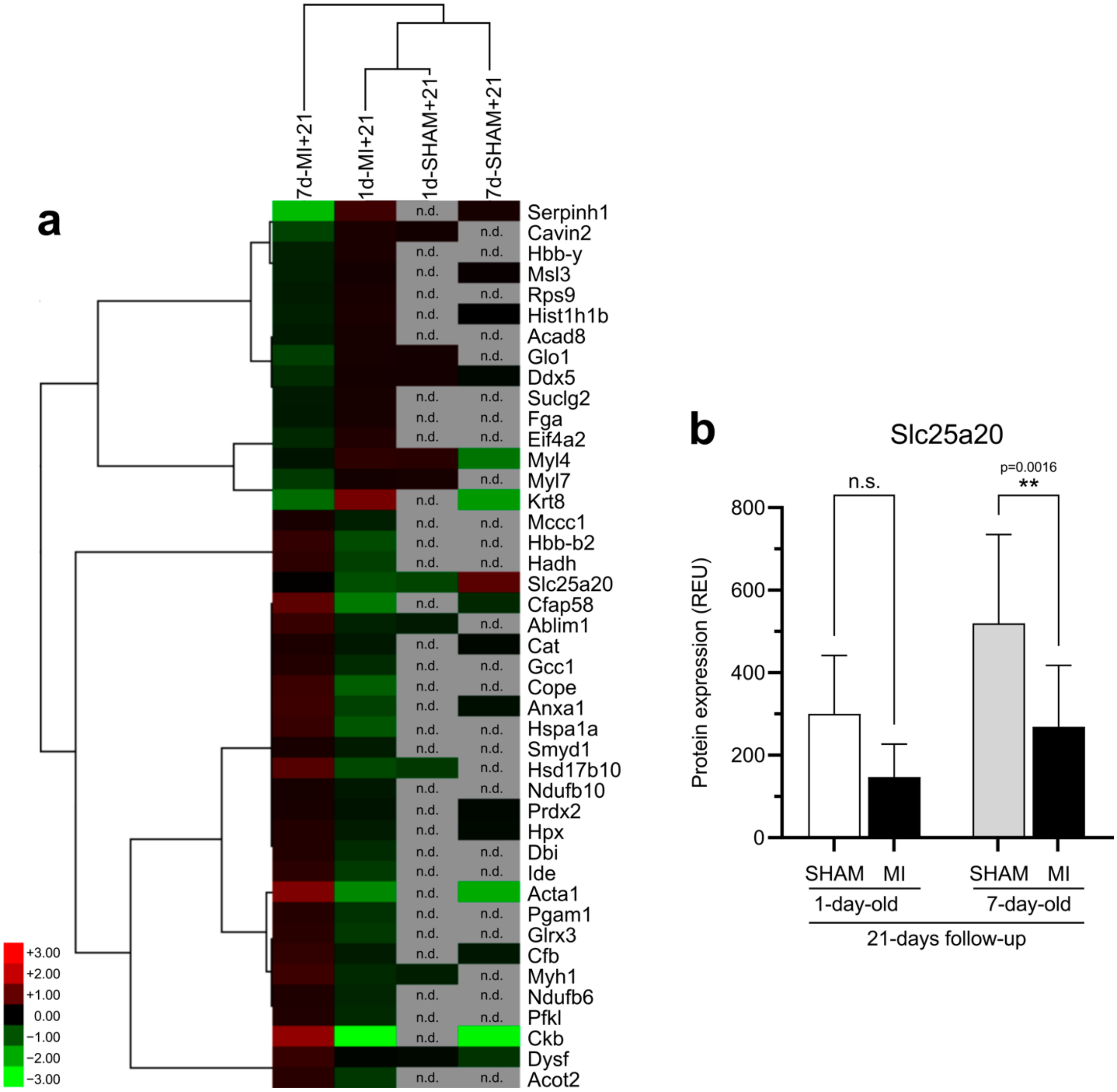

Supplementary Fig. 3. a. Heatmap of differentially expressed proteins evaluated by the proteomics approach in 1d regeneration-competent and 7d regeneration-compromised mice 21 days after myocardial infarction. **b.** Comparison of Slc25a20 expression in the proteomics analysis between groups 21 days after induction of myocardial infarction (MI) or sham operation (SHAM). ** p<0.01; REU, relative expression units.

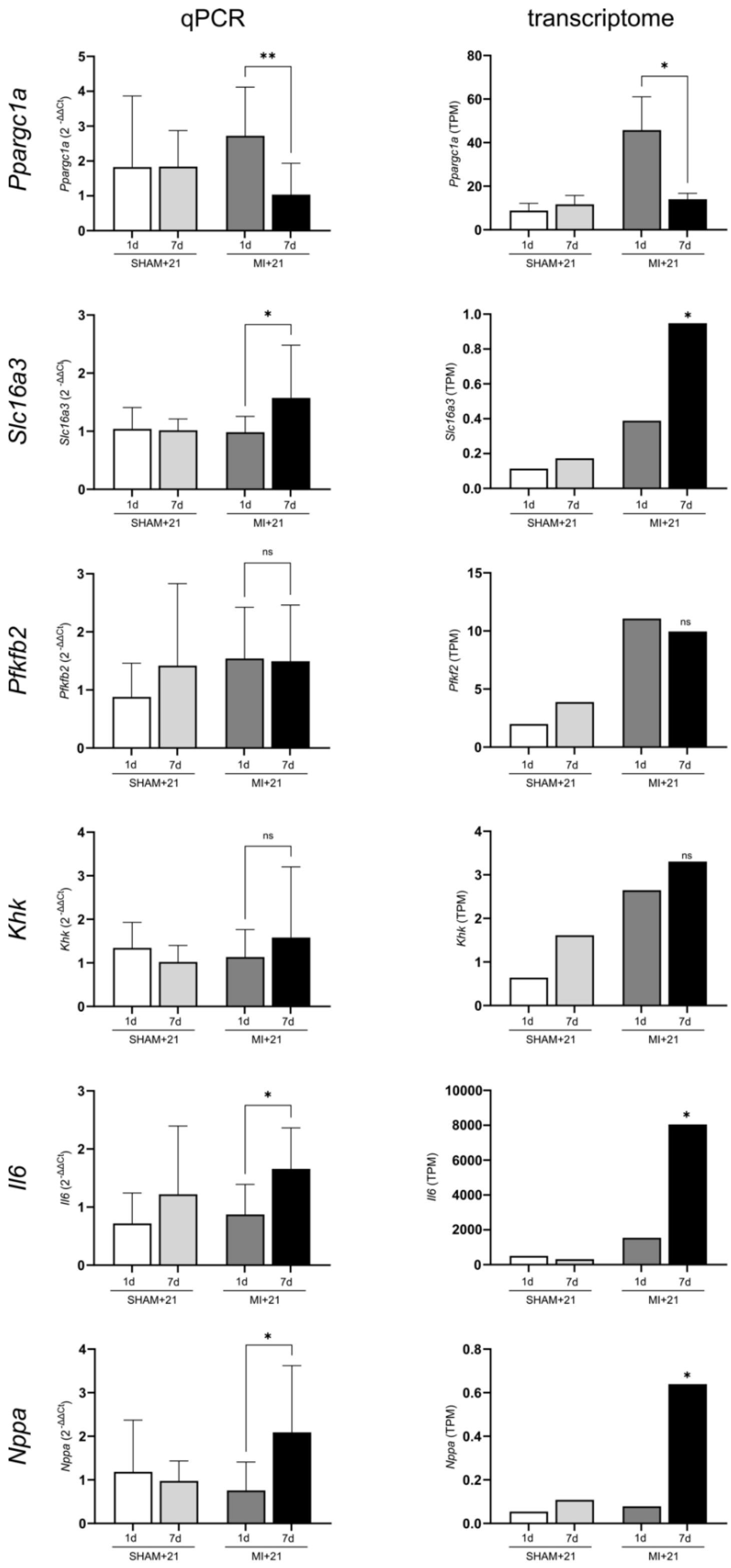

Supplementary Fig. 4. Validations of selected transcripts in the +21 differential transcriptome using qPCR **-** comparison with global transcriptome analysis approach.

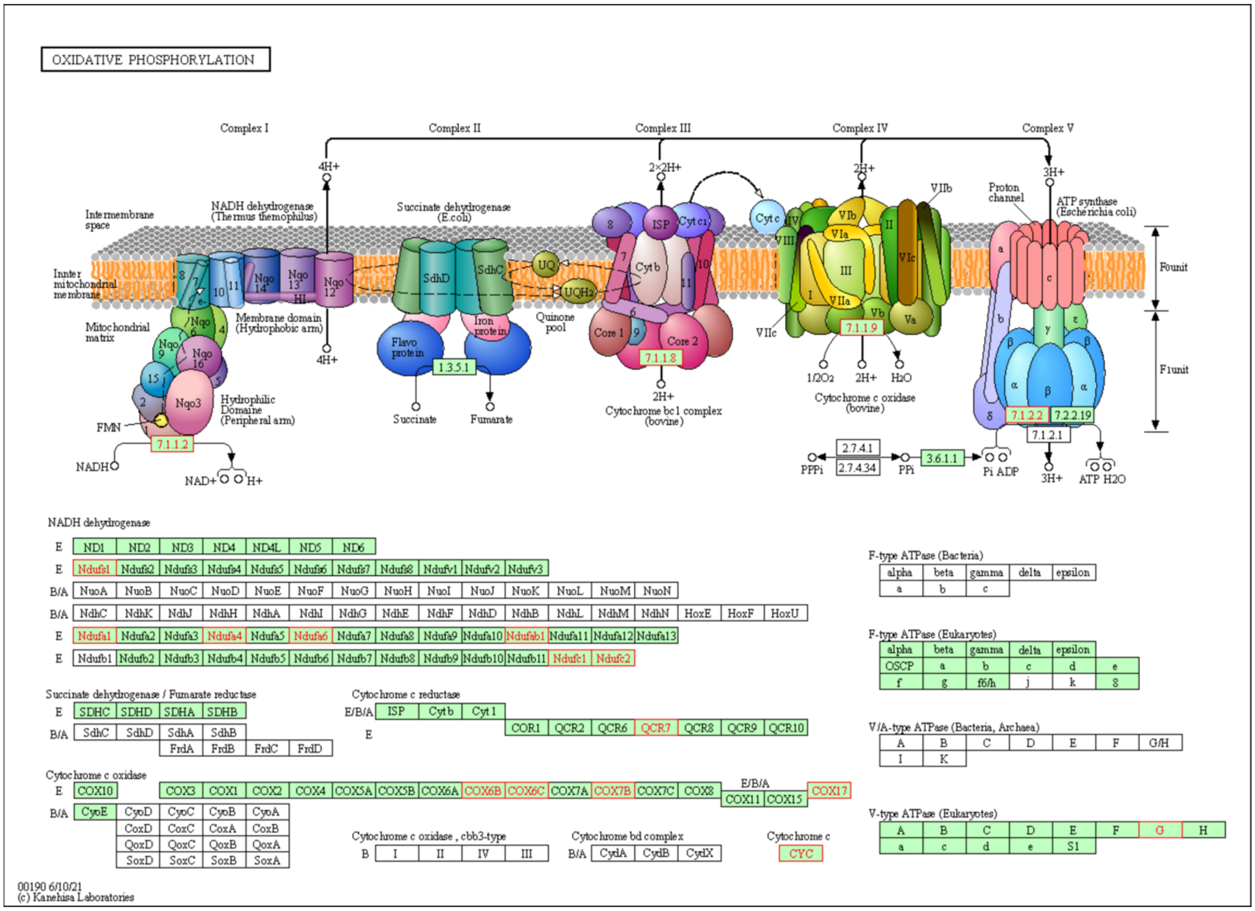

Supplementary Fig. 5. Differential comparisons of gene expression at 3 and 21 days after myocardial infarction on the mitochondrial electron transport chain according to the KEGG pathway Oxidative Phosphorylation (mmu00190). Red text and frames show the differentially expressed genes in the pathway. Please also see the Supplementary Table 5.

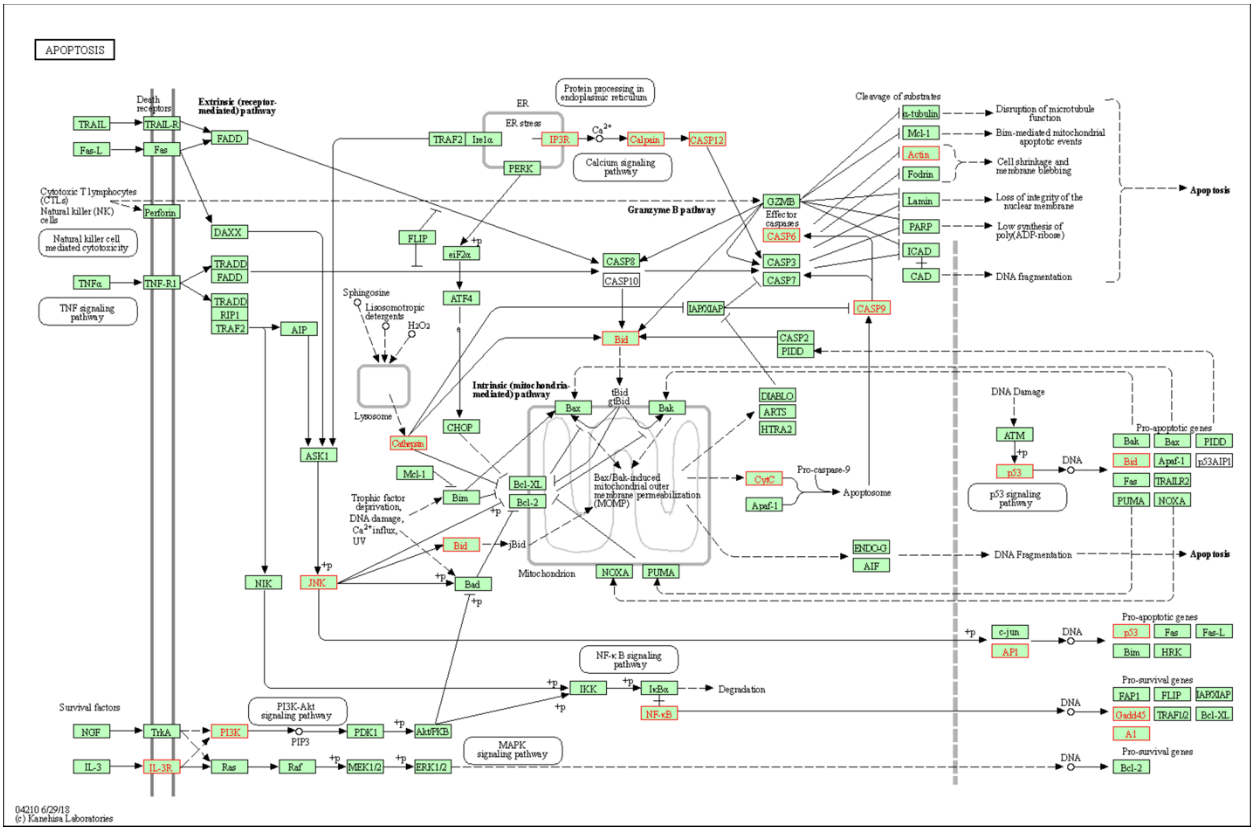

Supplementary Fig. 6. Differential comparisons of gene expression at 3 and 21 days after myocardial infarction on apoptosis according to the KEGG pathway Apoptosis (mmu04210). Red text and frames show the differentially expressed genes in the pathway. Please also see the Supplementary Table 6.

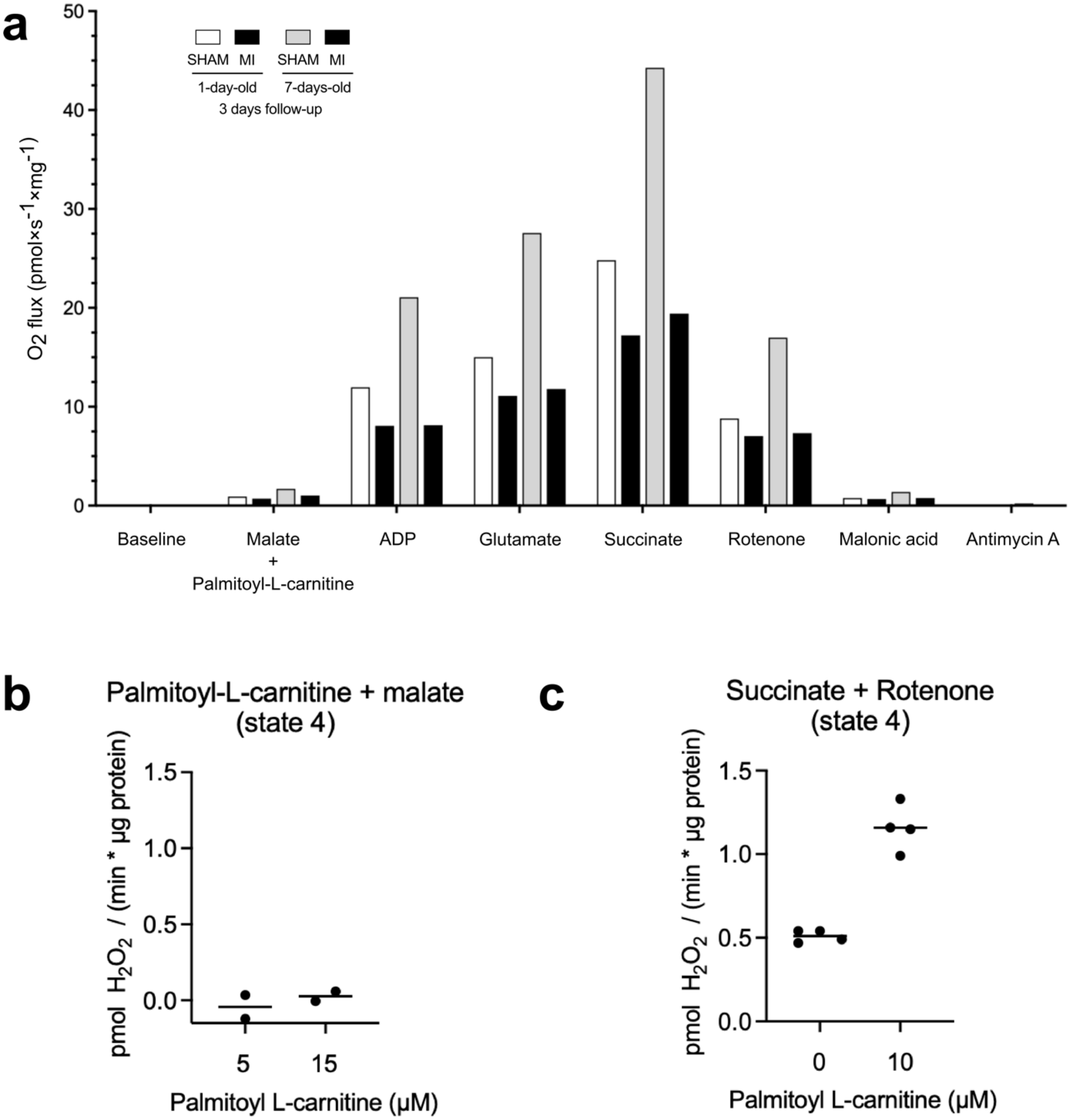

Supplementary Fig. 7. Analysis of beta-oxidation and OXPHOS, and modulation of mitochondrial ROS production by palmitoyl-L-carnitine. **a.** High-resolution respirometry assessment of mitochondria in the presence of 20 µM palmitoyl-L-carnitine. The substrates were added consecutively starting from the malate and palmitoyl-L-carnitine followed by ADP and the other indicated substrates. Malate was included to enable the entry of fatty acid-derived acetyl-CoA into the TCA cycle. Four hearts were pooled to generate a single pooled sample analysis. Data are presented as mean. **b.** Mitochondrial ROS production during palmitoyl-L-carnitine oxidation in isolated cardiac mitochondria of neonatal mice that did not undergo MI surgery. The malate concentration was 0.5 mM. **c.** Mitochondrial ROS production during succinate oxidation before and after the addition of palmitoyl-L-carnitine. Reverse-electron flow to complex I was blocked with 200 nM rotenone. Under these conditions palmitoyl-L-CoA does not undergo beta-oxidation due to mitochondrial acetyl-CoA and NADH accumulation. The succinate concentration was 10 mM. The ROS measurements were performed in the absence of ADP (state 4) to induce a high-membrane potential state, and thus to maximize ROS production at complex III (and at complex I in panel **b**).

Supplementary Methods

Animals

One- (P1) and seven-day (P7) old male Hsd:ICR(CD-1) mice were divided in four groups as follows: 1) 1-day-old sham operated (1d-SHAM+21d), 2) MI-induced 1-day old (1d-MI+21d), 3) 7-day-old sham-operated (7d-SHAM+21d), and MI-induced 7-day-old (7d-MI+21d). These animals were subjected to a follow up period of 21 days. A complementary study was made where the follow-up period was shortened to three days (1d-SHAM+3, 1d-MI+3, 7d-SHAM+3, 7d-MI+3). To carry out the necessary measurements from the small-sized hearts, a total of 193 animals were used. All animal experiments were carried out according to the European Community guidelines for use of experimental animals and approved by the Finnish National Animal Experiment Board (permission numbers ESAVI/8054/04.10.07/2016, ESAVI/31851/2019). The animal model of the study was chosen based on the pioneering work of Porrello *et al.*[1], who showed the different regenerative capacities between the P1 and P7 mice. Moreover, the chosen animal strain has been characterized as having large litters, excellent maternal characteristics and infrequent neonatal cannibalism (https://www.envigo.com/model/hsd-icr-cd-1).

Operations

The myocardial infarction model has been previously reported in detail by Mahmoud *et al.*[2] and Blom *et al.*[3]. In brief, one- and seven-day old male mice pups were removed from their maternal cage and were placed on a piece of sterile gauze lying on wet ice until slowing of breathing reflex and unresponsiveness for tail pinch was evident (from 2 to 3 min). After skin incision, thoracotomy was made through the 4th intercostal space with the aid of small scissors and forceps. Next, the left anterior descending coronary artery (LAD) was ligated with 10-0 prolene monofilament. Ischemia was visually confirmed as the myocardium distal from the suture became paler. The thoracal incision was sutured with 8-0 prolene, and the pups were carefully wiped clean and placed on a +37 °C heating pad until they regained consciousness. The pups were then moved into a heated chamber (+37 °C) holding their littermates with abundant bedding from their mother’s cage. After the operated littermates were adequately awake, they were returned to their mother for the duration of the follow-up period (21 days or 3 days).

Echocardiography

Transthoracic echocardiography was performed on post-operative days 1, 3, 7 and 21 by using a Vevo 2100 ultrasound with MS700 (bandwidth 30–70 MHz) linear array transducer (VisualSonics Inc., Toronto, Canada). The images were stored and analyzed using Vevo LAB 1.7.1 software (VisualSonics Inc., Toronto, Canada). The chamber diameter was measured from the 2D-guided parasternal short axis M-mode images from which ejection fraction (EF) and fractional shortening (FS) were calculated. Of all animals used in this study, a total of 109 were subjected to echocardiography (n=8–14).

Sample collection

At the end of the follow up period (3 days or 21 days), the animals were weighed, anesthetized with isoflurane and decapitated. The hearts were exposed, collected and weighed. The samples for conventional histology were collected as cross-sections of the whole heart distal from the LAD ligature. The samples for TEM, respirometry, RNASeq, miRNASeq, proteomics and metabolomics, in turn, were collected from the non-infarcted area of the left ventricle (border and remote zones). As the neonatal mouse heart is small, separate series for echocardiography and

histology (n=6–10), electron microscopy (n=3–4), cardiac volumetry (n=4), mitochondrial high-resolution respirometry and RNA sequencing (n=4), proteomic analysis (n=4) and metabolomic analysis (n=6) were carried out to ensure that enough representative sample was available for each analysis. Heart samples for histology were fixed in formalin, embedded in paraffin and processed to hematoxylin and eosin (HE)-stained sections with routine protocols. Samples (~1 mm^3) for transmission electron microscopy were collected from the non-infarcted area (remote zone) of the left ventricular wall, fixed in 2.5% glutaraldehyde in 100 mM sodium-phosphate buffer and subsequently embedded in resin by using the normal protocols. Samples for transcriptomics, proteomics and metabolomics were frozen in liquid nitrogen and stored at −80 °C until analysis. Following the samples collection, the gender of the pups was verified by gonadal examination.

Isolation of mitochondria

Neonatal hearts were pooled (from 3-4 mice) to obtain approximately 20 mg tissue. The heart tissue was minced with scissors in trypsin-EDTA phosphate-buffered saline solution for 15 min on ice. The trypsin digestion was stopped by addition of fetal bovine serum (10%). The tissue pieces were collected by centrifugation (200g, 1 min) and the buffer replaced to 225 mM mannitol, 75 sucrose, 1 mM EGTA, 0.1 mg/ml fatty-acid free BSA, and 10 mM Tris-Cl pH 7.4 (at 0^oC). Homogenization was performed with Wheaton 5 ml glass-teflon Potter-Elvehjem. Nuclei and unhomogenized material were pellet by a low-speed centrifugation (800g, 10 min at 4^oC). The supernatant was subjected to 7800g centrifugation (5 min at 4^oC) to obtain the mitochondrial fraction. The mitochondria were washed once with homogenization buffer lacking BSA and re-pelleted (7800g, 5 min).

High-resolution respirometry

The rate of oxidative phosphorylation (OXPHOS) after 3d and 21d of follow-up was evaluated using Oxygraph-2k instrument (OROBOROS Instruments Corp., Innsbruck, Austria), as previously described[4]. Briefly, less than 5 mg of myocardial tissue was homogenized under ice-cold conditions in a SG3 shredder (Pressure Biosciences Inc., South Easton, MA, USA) in tubes containing 500 µl MiRO6 buffer (0.5 mM EGTA, 3 mM $MgCl_2 \cdot 6H2O$, 60 mM K-lactobionate, 20 mM taurine, 10 mM KH_2PO_4, 20 mM HEPES, 110 mM sucrose, 1 g/l BSA, 280 U/ml catalase, pH 7.1). The sample was then further diluted with 4.5 ml of MiRO6 buffer and 2.5 ml transferred to each of the two chambers of an Oxygraph-2k instrument and allowed to incubate 20 min before starting the substrate-uncoupler-inhibitor titration (SUIT) protocol[5].

The rate of beta oxidation during the first 10 postnatal days was evaluated from the 3d follow-up groups on the Oxygraph-2k by using isolated pooled cardiac mitochondria from 2–4 hearts (22.1 mg–26.9 mg). After isolation, mitochondria were suspended in MiRO6 buffer. From this suspension, 20 µl were transferred to an Oxygraph-2k instrument chamber prefilled with 2.5 ml MiRO6. The fatty acid protocol[6] was used with minor modifications as published previously[7]. Substrates and inhibitors were pipetted in the following order: (1) malate (2 mM)+palmitoyl-L-carnitine (20µM), (2) ADP+Mg^{2+} (1.25 mM), (3) glutamate (10 mM), (4) succinate (10 mM), (5) rotenone (0.2 µM), (6) malonic acid (5 µM), (8) antimycin a (2.5 µM). Results were standardized per mg of tissue wet weight.

Measurement of reactive oxygen species (ROS) production

Amplex UltraRed-peroxidase assay was employed to measure ROS production from isolated heart mitochondria. The measurements were performed at 37ºC in 96-well microplate format with a reaction volume of 0.3 ml and 12.5 µg mitochondrial protein. Mir05 buffer (Mir06 lacking catalase) served as the assay media. The Amplex UltraRed and horseradish peroxidase concentrations were 10 µM and 1 U/ml, respectively. Superoxide dismutase (5 U/ml) were included to ensure immediate dismutation of superoxide to H_2O_2. Catalase and thioredoxin system in samples were inhibited by 100 µM 3-amino-1,2,4-triazole and 0.5 µM auranofin. Background was determined in the presence of sample but in the absence of exogenous substrates required for the mitochondrial respiratory activity. Parallel reactions were spiked with a 50 pmol bolus of H_2O_2 (ε240 nm=43.6 $M^{-1}cm^{-1}$) to calibrate and convert the fluorescence signal (530 nm excitation, 590 nm emission) to the amount of H_2O_2 produced. The incubations were kept short (< 6 min) to avoid fluctuation in oxygen concentration.

Whole-mount immunostaining and volumetric microscopy

For whole-mount immunostaining, the animals were perfused with saline to remove excess blood and then perfusion-fixed with 10% paraformaldehyde. Hearts were removed and kept in 10% paraformaldehyde solution for 24 hours until stored in methanol. Immunolabeling and tissue clearing process was carried out with the iDISCO method out as described previously in detail by Renier et al.[8]. Cardiac Troponin C (ab30807, dilution 1:500, Abcam, Cambridge, United Kingdom) antibody was used for immunolabeling of cardiomyocytes. Images were captured by using a Bioptonics OPT Scanner 3001 M (LifeArc, London, United Kingdom) and 3D reconstructions from the data were made using NRecon software (LifeArc). Segmentation and analysis of MI size was made with Imaris software (Bitplane AG, Zürich, Switzerland). Briefly, MI size was calculated as a proportion of viable myocardium recognized with Troponin C antibody compared to whole heart quantified with visible light channel.

General histology and mitochondrial ultrastructure

Histology of the myocardial samples was evaluated in a blinded fashion with conventional light microscopy. In brief, histopathology of the arteries and cardiac muscle tissue was examined. The severity of the observed lesions in the myocardium, including tissue necrosis, fibrosis, and inflammatory cell infiltration, was graded with numerical values denoting to the degree of damage at the whole tissue level. The following system of severity scoring was used: 0, no abnormalities detected; 1, minimal; 2, mild; 3, moderate; 4, marked; or 5, severe[9, 10]. For the transmission electron microscopy, thin-sectioned samples were viewed and photographed with a Jeol JEM-1400 transmission electron microscope (Jeol Ltd., Tokyo, Japan). Mitochondrial number, volume fraction and trans-sectional area were analyzed with ImageJ software[11] (v1.47). For the number of cristae per µm, the ImageJ line tool was used to draw a line across the stack of cristae, perpendicular to their orientation. The number of cristae crossing the line was counted and expressed in relation to the line length[12].

Global metabolomics analysis

Metabolomics analysis was carried out as previously described[4]. Hearts from the SHAM and MI-treated groups collected after 21-day follow-up period were shipped to Metabolon, Inc (Durham, NC, USA) where they were stored at −80 °C until analysis. Sample preparation, quality control, UPLC-MS/MS and data analysis were all carried out by Metabolon, Inc. Samples were

prepared using the automated MicroLab STAR system with standards for QC (Hamilton, Reno, NV, USA). Proteins were precipitated with methanol followed by shaking and centrifugation. The extract was divided into following fractions: two for analysis by two separate reverse-phase (RP)/UPLC-MS/MS methods with positive ion mode electrospray ionization (ESI), one for analysis by RP/UPLC-MS/MS with negative ion mode ESI, and one for analysis by HILIC/UPLC-MS/MS with negative ion mode ESI. All methods utilized a Waters ACQUITY ultra-performance liquid chromatography (UPLC) with Waters (UPLC BEH C18-2.1×100 mm, 1.7 µm and BEH Amide 2.1x150 mm, 1.7 µm) columns and a Thermo Scientific Q-Exactive mass spectrometer interfaced with a heated electrospray ionization (HESI-II) source and Orbitrap mass analyzer operated at 35,000 mass resolution[13]. Metabolites were identified by comparison to library entries of purified standards or recurrent unknown entities based on authenticated standards that contains the retention time/index (RI), mass to charge ratio (*m/z*), and chromatographic data (including MS/MS spectral data) on all molecules present in the library[14]. Peaks were quantified using area-under-the-curve. The data underwent random forest analysis, and two-way Anova tests followed by Welch's two-sample t-test according to the statistics workflow of Metabolon Inc. were used to identify molecules that differed significantly between experimental groups ($p \leq 0.05$).

Transcriptomics analysis

The cardiac samples for whole transcriptome and miRNA analysis were removed from −80 °C and processed with the Precellys 24 homogenizer with ceramic beads (Bertin Technologies, Montigny-le-Bretonneux, France). RNA was isolated with TRIzol reagent, followed by DNAse I treatment. Agilent Bioanalyzer RNA pico chip (Agilent Technologies Inc., Santa Clara, CA, USA) was used to evaluate the integrity of RNA and Qubit RNA-kit (Thermo Fisher Scientific Inc., Waltham, MA, USA) to quantitate RNA.

mRNA analysis

Library preparation and NGS of samples collected after 21 days of follow-up were made by the Institute for Molecular Medicine Finland (FIMM), University of Helsinki, Finland. rRNA depletion was done according to RiboZero magnetic Gold protocol (Illumina Inc.) and the library preparation was done by using ScriptSeqV2 library preparation kit Library Prep Kit (Illumina Inc.). The libraries were quantified and pooled for sequencing based on Agilent Bioanalyzer HiSens kit. Sequencing was performed on Illumina HiSeq 2000 instrument (2x100 cycles), according to the manufacturer instructions. Library preparation and next generation sequencing (NGS) of samples collected at 3 days follow-up were made with TruSeq stranded total RNA Library Prep Kit and NextSeq550 instrument (Illumina Inc., San Diego, CA, USA) by QIAGEN Sciences Inc. (Germantown, MD, USA, http://www.qiagen.com). The starting material (500 ng) of total RNA was rRNA depleted using biotinylated, target-specific oligos combined with Ribo-Zero rRNA removal beads. The isolated RNA was subsequently fragmented using enzymatic fragmentation. Then first and second strand synthesis were performed, and the double stranded cDNA was purified (AMPure XP, Beckman Coulter Inc., Brea, CA, USA). The cDNA was end-repaired, 3'-adenylated and Illumina sequencing adaptors ligated onto the fragments ends, and the library was purified (AMPure XP). The RNA stranded libraries were pre-amplified with PCR and purified (AMPure XP). The libraries size distribution was validated, and quality inspected on a Bioanalyzer 2100 or BioAnalyzer 4200 TapeStation (Agilent Technologies). High quality libraries were pooled in equimolar concentrations based on the Bioanalyzer Smear Analysis tool (Agilent Technologies). The library pool(s) were quantified using qPCR and optimal concentration of the library pool used

to generate the clusters on the surface of a flow cell before sequencing on a NextSeq550 instrument (2x75 cycles), according to the manufacturer instructions (Illumina Inc.).

The bioinformatic analysis of NGS data was carried out with "Tuxedo" software consisting of Bowtie2 (v. 2.2.2), Tophat (v2.0.11), Cufflinks (v2.2.1; Qiagen) including alignment of reads to the mouse reference genome (GRCm38; Ensembl_81), feature summation, and normalization of expression estimates. To allow for the comparison of differential expression estimates between samples, the normalized expression values for each transcript were calculated as Fragments per Kilobase per Million Mapped Reads (FPKM) with Cuffdiff2. The expression profiles of different treatment groups were compared and the transcript ratios between the average FPKMs were calculated. Transcripts showing a LogFC ≥1 and a False Discovery Rate (FDR) <0.05 were classified as differentially expressed.

miRNA analysis

Library preparation and NGS of samples collected at the 21-day follow-up were made by the Institute for Molecular Medicine Finland (FIMM), University of Helsinki, Finland. The library preparation was done using the TruSeq Small RNA Sample Prep kit (Illumina Inc., San Diego, CA, USA). The small RNA fractions of the libraries were enriched by using Caliper XT system (PerkinElmer, Waltham, MA, USA) Caliper XT 300 DNA chip was used and 147bp fragments were selected for the enrichment. The size selected libraries were quantified for sequencing based on Agilent Bioanalyzer HiSens kit. Sequencing was performed on Illumina HiSeq 2000 instrument (1x100 cycles), according to the manufacturer instructions (Illumina Inc., San Diego, CA, USA) Annotation of the obtained sequences was performed by using the reference annotation (GRCm38; miRbase_20). Library preparation and NGS of samples collected at 3-days follow-up were made by QIAGEN Sciences Inc. using QIAseq miRNA Library Kit (QIAGEN Sciences Inc.) and NextSeq500 instrument (Illumina Inc.) by QIAGEN Sciences Inc. The library preparation was done using the QIAseq miRNA Library Kit (Qiagen). A total of 100ng total RNA was converted into miRNA NGS libraries. Adapters containing UMIs were ligated to the RNA. Then RNA was converted to cDNA. The cDNA was amplified using PCR (16 cycles) and during the PCR, indices were added. After PCR, the samples were purified. Library preparation QC was performed using either Bioanalyzer 2100 (Agilent) or TapeStation 4200 (Agilent). Based on quality of the inserts and the concentration measurements, the libraries were pooled in equimolar ratios. The library pool(s) were quantified using qPCR. The library pool(s) were then sequenced on a NextSeq500 sequencing instrument according to the manufacturer instructions.

The data was UMI corrected and mapped (Bowtie2 v.2.2.2.). Annotation of the obtained sequences was performed by using the reference annotation (GRCm38; miRbase_20). Differential expression analysis was performed using the EdgeR statistical software package (Bioconductor, http://bioconductor.org/). For normalization, the trimmed mean of M-values method based on LogF and absolute gene-wise changes in expression levels between samples (TMM normalization) were used. Transcripts showing a LogFC ≥1 and a False Discovery Rate (FDR) <0.05 were classified as differentially expressed.

Proteomics analyses

Hearts from the 1d-MI+21 and 7d-MI+21 and their corresponding controls were homogenized at 4 °C in 7M urea, 2M thiourea, 4% CHAPS using a Precellys® 24 homogenizer (Bertin Technologies) with ceramic beads. Samples were centrifuged for 1 minute at 16.000 g. 10 μg of total protein lysates were processed using modified FASP protocol as described[15]. The

samples were analyzed by DIA-HDMS^E (data independent acquisition high-definition ion-mobility enabled tandem mass spectrometry), as described[4].

Relative quantification between samples using precursor ion intensities was achieved with Progenesis QI for ProteomicsTM Informatics for Proteomics software (Nonlinear Dynamics/ Waters Corporation, Milford, MA, USA) and ProteinLynx Global Server (PLGS v3.0)[16]. Label-free protein quantitation method was applied for post processing data analysis using precursor ion intensity data and standardized expression profiles. Database searches were carried out against *Mus musculus* (release 2018_16993 entries) UniProtKB/SwissProt, reviewed database (with primary digest reagent set for trypsin, 2 missed cleavages allowed, cysteine residue carbamidomethylation as fixed modification and as variable modifications, deamidation of asparagine and glutamine residues and oxidation of methionine). Differentially expressed proteins (DEP) were identified based on the number of unique peptides used for label-free quantitation (≥2), at the FDR<0.01 and the fold change (FC) ratio from averaged, normalized protein intensities |FC≥1.5|, and p≤ 0.05 by ANOVA in all comparisons.

The lists of up/down regulated protein changes with their corresponding, unique UniProtKB-database identifiers served as inputs into Ingenuity Pathways (IPA, Ingenuity systems, Redwood City, CA, USA; www.ingenuity.com), with a focus on Canonical pathways, and Disease and Function annotation.

Ingenuity Pathway Analysis (IPA)

Pathway analyses were carried out as described earlier[17–19]. Briefly, pre-filtered lists of differentially expressed genes were imported into and analyzed using the IPA software (Qiagen). Analysis of miRNA utilized the IPA miRNA filter and only miRNAs' and their target genes' expressions at opposite directions were considered meaningful as miRNA-dependent regulation. Exploratory analysis and hypothesis-generating network building included genes with differential $|\log_2|$ expression fold changes greater than 1 between the 1d-MI+21 and 7d-MI+21 groups and one-sided t-test p values less than 0.05. The top-scoring, differentially expressed genes identified were then evaluated using R Studio (2021.09.1+372 "Ghost Orchid" http://www.rstudio.com/) Benjamini-Hochberg FDR-controlling method between groups. For visualization, the String network was imported into Cytoscape 3.9.0[20]. Visualization used Nested network style and yFiles Organic layout under academic licensing[21] (http://www.yworks.com). Final graphical layout design of the networks was carried out in Inkscape (1.0.1; https://inkscape.org).

Quantitative RT-PCR (qRT-PCR)

The hearts from 1-day-old and 7-day-old mice were snap-frozen in liquid nitrogen and processed with the Precellys 24 homogenizer with ceramic beads (Bertin Technologies, Montigny-le-Bretonneux, France). RNA from the homogenized samples was isolated with TRIzol as described[22], followed by DNAse I treatment. Agilent Bioanalyzer RNA pico chip (Agilent Technologies, Santa Clara, CA, USA) was used to evaluate the integrity of RNA and Qubit RNA-kit (Life Technologies, Carlsbad, CA, USA) to quantitate RNA.

The RNA was reverse-transcribed using Bioline SensiFAST cDNA synthesis kit (Meridian Bioscience Inc., Cincinnati, OH, USA) according to the manufacturer's instructions. Primer sequences of qPCR primers were as follows: Khk-c 5'-GCGTGGATGTGTCTCAGGTG-3' and 5'-TGTTGACGATGCAGCAAGA-3', Pfkfb2 5'-CGGGAATGGATCTACACTGG-3' and 5'-GGAGAGCAAAGTGAGGGATG-3', Slc16a1 5'-GCTGGAGGTCCTATCAGCAG-3' and 5'-

AGTTGAAAGCAAGCCCAAGA-3', Il6 5'-TGATGCACTTGCAGAAAACA-3' and 5'-ACCAGAGGAAATTTTCAATAGGC-3', Ppargc1a 5'-CATGTGCAGCCAAGACTCTG-3' and 5'-ACACCACTTCAATCCACCCA-3', Nppa 5'-GAAAAGCAAACTGAGGGCTCTG-3´ and 5'-CCTACCCCCGAAGCAGCT-3', and 18S 5'-ACATCCAAGGAAGGCAGCAG-3' and 5'-TTTTCGTCACTACCTCCCCG-3'. Quantitative real-time PCR reactions were prepared with Roche FastStart Essential DNA Green Master as recommended by the manufacturer and run on a LightCycler 96 instrument (Roche Molecular Systems Inc., Pleasanton, CA, USA). Ct-values were normalized to the housekeeping gene 18S. Relative gene expression values were calculated as described[23].

References to Supplementary Materials

1. Porrello, E.R. *et al.* Transient regenerative potential of the neonatal mouse heart. *Science.* **331**, 1078–1080 (2011).

2. Mahmoud, A.I., Porrello, E.R., Kimura, W., Olson, E.N. & Sadek HA. Surgical models for cardiac regeneration in neonatal mice. *Nat. Protoc.* **9,** 305–311 (2014).

3. Blom, J.N., Lu, X., Arnold, P. & Feng, Q. Myocardial Infarction in Neonatal Mice, A Model of Cardiac Regeneration. *J. Vis. Exp.* **111**, 54100 (2016).

4. Lalowski, M.M. *et al.* Characterizing the Key Metabolic Pathways of the Neonatal Mouse Heart Using a Quantitative Combinatorial Omics Approach. *Front. Physiol.* **9**, 365 (2018).

5. Pesta, D. & Gnaiger, E. High-resolution respirometry: OXPHOS protocols for human cells and permeabilized fibers from small biopsies of human muscle. *Methods Mol. Biol.* **810**, 25–58 (2012).

6. Lemieux, H., Semsroth, S., Antretter, H., Höfer, D. & Gnaiger, E. Mitochondrial respiratory control and early defects of oxidative phosphorylation in the failing human heart. *Int. J. Biochem. Cell Biol.* **43**, 1729–1738 (2011).

7. Kivelä, R. *et al.* VEGF-B-induced vascular growth leads to metabolic reprogramming and ischemia resistance in the heart. *EMBO Mol. Med.* **6**, 307–321 (2014).

8. Renier, N., Wu, Z., Simon, D.J., Yang, J., Ariel, P. & Tessier-Lavigne, M. iDISCO: a simple, rapid method to immunolabel large tissue samples for volume imaging. *Cell.* **159**, 896–910 (2014).

9. Herbert, R.A., Hailey, J.R. & Seely, J.C. Nomenclature. In Handbook of Toxicologic Pathology, Haschek, W.M., Rousseaux C.G. & Wallig, M.A. (eds). San Diego, Academic Press, pp 157–167 (2002).

10. Mann, P.C. *et al.* International harmonization of toxicologic pathology nomenclature: an overview and review of basic principles. *Toxicol. Pathol.* **40 (4 Suppl)**, 7S–13S (2012).

11. Schneider, C.A., Rasband, W.S. & Eliceiri, K.W. NIH Image to ImageJ: 25 years of image analysis. *Nat. Methods.* **9**, 671–675 (2012).

12. Puente, B.N. *et al.* The oxygen-rich postnatal environment induces cardiomyocyte cell-cycle arrest through DNA damage response. *Cell.* **157**, 565–579 (2014).

13. Evans, A.M., DeHaven, C.D., Barrett, T., Mitchell, M. & Milgram, E. Integrated, nontargeted ultrahigh performance liquid chromatography/electrospray ionization tandem mass spectrometry platform for the identification and relative quantification of the small-molecule complement of biological systems. *Anal. Chem.* **81**, 6656–6667 (2009).

14. Dehaven, C.D., Evans, A.M., Dai, H. & Lawton, K.A. Organization of GC/MS and LC/MS metabolomics data into chemical libraries. *J. Cheminform.* **2**, 9 (2010).

15. Scifo, E. *et al.* Proteomic analysis of the palmitoyl protein thioesterase 1 interactome in SH-SY5Y human neuroblastoma cells. *J. Proteomics.* **123**, 42–53 (2015).

16. Laakkonen, E.K. *et al.* Estrogenic regulation of skeletal muscle proteome: a study of premenopausal women and postmenopausal MZ cotwins discordant for hormonal therapy. *Aging Cell.* **16**, 1276–1287 (2017).

17. Lindford, A. *et al.* Case Report: Unravelling the Mysterious Lichtenberg Figure Skin Response in a Patient With a High-Voltage Electrical Injury. *Front. Med. (Lausanne).* **8**, 663807 (2021).

18. Mulari, S. *et al.* Ischemic Heart Disease Selectively Modifies the Right Atrial Appendage Transcriptome. *Front. Cardiovasc. Med.* **8,** 728198 (2021).

19. Klaas, M. *et al.* Thrombospondin-4 Is a Soluble Dermal Inflammatory Signal That Selectively Promotes Fibroblast Migration and Keratinocyte Proliferation for Skin Regeneration and Wound Healing. *Front. Cell Dev. Biol.* **9**, 745637 (2021).

20. Shannon, P. *et al.* Cytoscape: a software environment for integrated models of biomolecular interaction networks. *Genome Res.* **13**, 2498–2504 (2003).

21. Wiese, R., Eiglsperger, M. & Kaufmann, M. yFiles: Visualization and Automatic Layout of Graphs. Berlin, Heidelberg: Springer, pp. 453–454 (2002).

22. Rio, D.C., Ares, M. Jr., Hannon, G.J. & Nilsen, T.W. Purification of RNA using TRIzol (TRI reagent). *Cold Spring Harb. Protoc.* **2010**, pdb.prot5439 (2010).

23. Livak, K.J. & Schmittgen, T.D. Analysis of relative gene expression data using real-time quantitative PCR and the 2(-Delta Delta C(T)) Method. *Methods.* **25**, 402–408 (2001).
